# Metabolic Profiling, Antioxidant Activity, and Mechanistic Insights of Chicory from Northern and Southern China: Integrating Metabolomics, Machine Learning, Molecular Docking, Molecular Dynamics Simulations, and In Vitro Assays

**DOI:** 10.3390/molecules31203790

**Published:** 2026-10-21

**Authors:** Dan He, Yaxuan Li, Jiarun Zhang, Qilin Yang, Wanjia Lian, Jin Li, Lin Yang

**Affiliations:** 1 College of Chinese Materia Medica, Chongqing College of Traditional Chinese Medicine, Chongqing 402760, China; hedan@cqctcm.edu.cn (D.H.); 18725768058@139.com (J.Z.); 13527413980@163.com (Q.Y.); 18990607243@163.com (J.L.); 2 iHuman Institute, ShanghaiTech University, Shanghai 201210, China; 18099910025@163.com; 3 Center for Technology Industry Development, Chongqing Medical and Pharmaceutical College, Chongqing 401331, China

**Keywords:** chicory, geographical variation, metabolomics, machine learning, molecular dynamics simulation, antioxidant activity

## Abstract

Chicory is a traditional food–medicinal plant, and its antioxidant efficacy has been recognized. However, the specific differences in the metabolic characteristics and antioxidant activity of chicory caused by geographical differences remain unclear. In this study, a total of 64 volatile components and 35 non-volatile components of northern and southern chicory were identified. Chemometric combined with machine learning was applied to screen 8 volatile and 7 non-volatile potential chemical biomarkers. Furthermore, the core targets of the chicory antioxidant were revealed by network pharmacology prediction combined with molecular docking and molecular dynamics simulation. The results of in vitro antioxidant assays illuminated that the antioxidant activity of northern chicory was significantly stronger than that of the southern samples (*p* < 0.01). This study reveals that geographical environments may affect the metabolites and antioxidant activity in chicory by regulating secondary metabolism, and it also provides a scientific basis for the application of chicory as a natural antioxidant in functional foods.

## 1. Introduction

Chicory refers to the dried aerial parts or roots of *Cichorium glandulosum* Boiss. et Huet or *Cichorium intybus* L., both belonging to the family Asteraceae. The aerial parts are harvested and cut during summer and autumn, while the roots are dug in late autumn. Thereafter, sediment and impurities are removed, and the materials are subjected to sun-drying [1]. Chicory is a perennial herb of the Asteraceae family, characterized by a well-developed taproot system, erect stems (1–1.5 m in height), lanceolate leaves, and blue capitula (rarely white or pink variants) [2]. It originates from the Mediterranean region, Central Asia, and North Africa. In the food field, the whole plant can be consumed as a vegetable, and its roots, after grinding and roasting, are often used as a coffee adjunct. It is rich in dietary fiber, inulin, as well as bioactive substances with antibacterial and antioxidant activities such as cinnamic acid and flavonoids. These components not only endow it with food application value but also enable it to exhibit certain medicinal development potential [3,4,5]. To summarize, chicory centers on its abundant food application value while also possessing medicinal potential; this food–medicinal dual property makes it have important application prospects in the food industry and functional product development.

Oxidative stress refers to a stress state caused by the imbalance between oxidation and antioxidant systems due to excessive production of reactive oxygen species (ROS) in the body upon stimulation [6]. Excessive accumulation of ROS not only drives physiological processes such as aging but also mediates the occurrence and development of various pathological conditions, including cancer, cardiovascular diseases, and inflammatory diseases [7,8]. Therefore, inhibiting excessive ROS production is crucial for maintaining cellular redox homeostasis and ensuring the normal physiological functions of living systems. Due to potential health risks associated with synthetic antioxidants [9], natural antioxidants have become a research focus owing to their definite efficacy and low toxicity. Among them, plant-derived components and monomers with strong antioxidant activity play significant roles in the prevention and treatment of oxidative stress-related diseases [10,11,12].

The Qinling–Huaihe Line, stretching west from the Qinling Mountains to the main stream of the Huaihe River, roughly traverses 33° N and serves as the geographical boundary between northern and southern China, with significant differences in climate, topography, and soil between the two regions [13]. Southern China has a subtropical monsoon climate, characterized by mild winters with less precipitation and hot summers with abundant rainfall, and the soil is dominated by acidic red soil. Northern China has a temperate monsoon climate, with cold and dry winters and hot and humid summers, and the soil is mostly alkaline, forming a sharp contrast with the southern region [14]. Studies have shown that environmental differences can alter the chemical composition and biological properties of plants [15,16,17]. Chicory is native to northern China (e.g., Xinjiang, Heilongjiang, Liaoning) and has been traditionally used in ethnic medicine by the Uyghur and Mongolian peoples. Since the 1980s, broad-leaf cultivars have gradually expanded to the southern regions [18]. The significant differences in climate and soil between northern and southern China may lead to spatial variations in the synthesis and accumulation of volatile and non-volatile components in chicory, thereby affecting its processing characteristics and edible value.

Metabolomics has emerged as a powerful tool for revealing differences in chemical component contents and the relationships between compounds and pharmacological effects. Metabolomics enables a comprehensive analysis of compounds in plants, thereby making it particularly valuable for screening potential biomarkers in traditional Chinese medicine [19]. Machine learning, the core of artificial intelligence, typically involves developing predictive models and identifying data patterns. In contrast to conventional statistical methods, machine learning offers numerous distinct advantages. Specifically, machine learning exhibits superior performance in addressing complex non-linear relationships and demonstrates high efficiency in processing large-scale datasets. Furthermore, machine learning is equipped with advanced evaluation index systems and the capability for a precise optimization of model performance [20,21]. Currently, the analytical approach combining machine learning and metabolomics has been applied in traditional Chinese medicine and food analysis, including the prediction of different grades of *Astragalus membranaceus*, the discrimination of geographical origins of cinnamon and Wuyi rock tea, and the classification of mung beans from different climates and cultivation regions [22]. Simultaneously, the performance of machine learning algorithms varies depending on specific samples and datasets, and different machine learning algorithms exhibit distinct performances within the same dataset.

Existing studies have identified components such as phenolics, carbohydrates, flavonoids, sesquiterpene lactones, volatile oils, and amino acids in chicory [23], but most focus on component comparisons within a single producing area or among different plant parts [24,25], with insufficient attention to geographical origin-induced component differentiation. Therefore, this study aims to systematically explore the quality differences and material basis of Chicory from different producing areas: first, headspace–gas chromatography–mass spectrometry (HS-GC-MS) and ultra-performance liquid chromatography–quadrupole time-of-flight mass spectrometry (UPLC-Q-TOF-MS) were used to identify volatile and non-volatile metabolites of Chicory from the northern and southern producing areas. Subsequently, chemometrics integrated with machine learning was employed to elucidate the regional differentiation patterns of metabolic composition and identify the key chemical biomarkers underlying the north–south divergence in *Cichorium intybus*. On this basis, network pharmacology was used to predict potential targets of active components, and molecular docking and molecular dynamics simulations were performed to verify their interaction mechanisms; finally, in vitro antioxidant assays were conducted to clarify the activity differences among chicory from different producing areas and their association with key components. This study, based on the combined approach of metabolomics and machine learning, enables the rapid and effective identification of differential components in northern and southern *Cichorium intybus* samples. Verified through molecular dynamics simulations and in vitro experiments, the material basis underlying the differences in antioxidant activity of *Cichorium intybus* from distinct producing regions was elucidated. Therefore, this research aims to provide a scientific basis for the precise application of chicory as a natural antioxidant in the screening and development of functional food ingredients.

## 2. Results and Discussion

### 2.1. GC-MS Analysis of Chicory from Different Regions

#### 2.1.1. Identification of Volatile Metabolites

**Figure 1:**
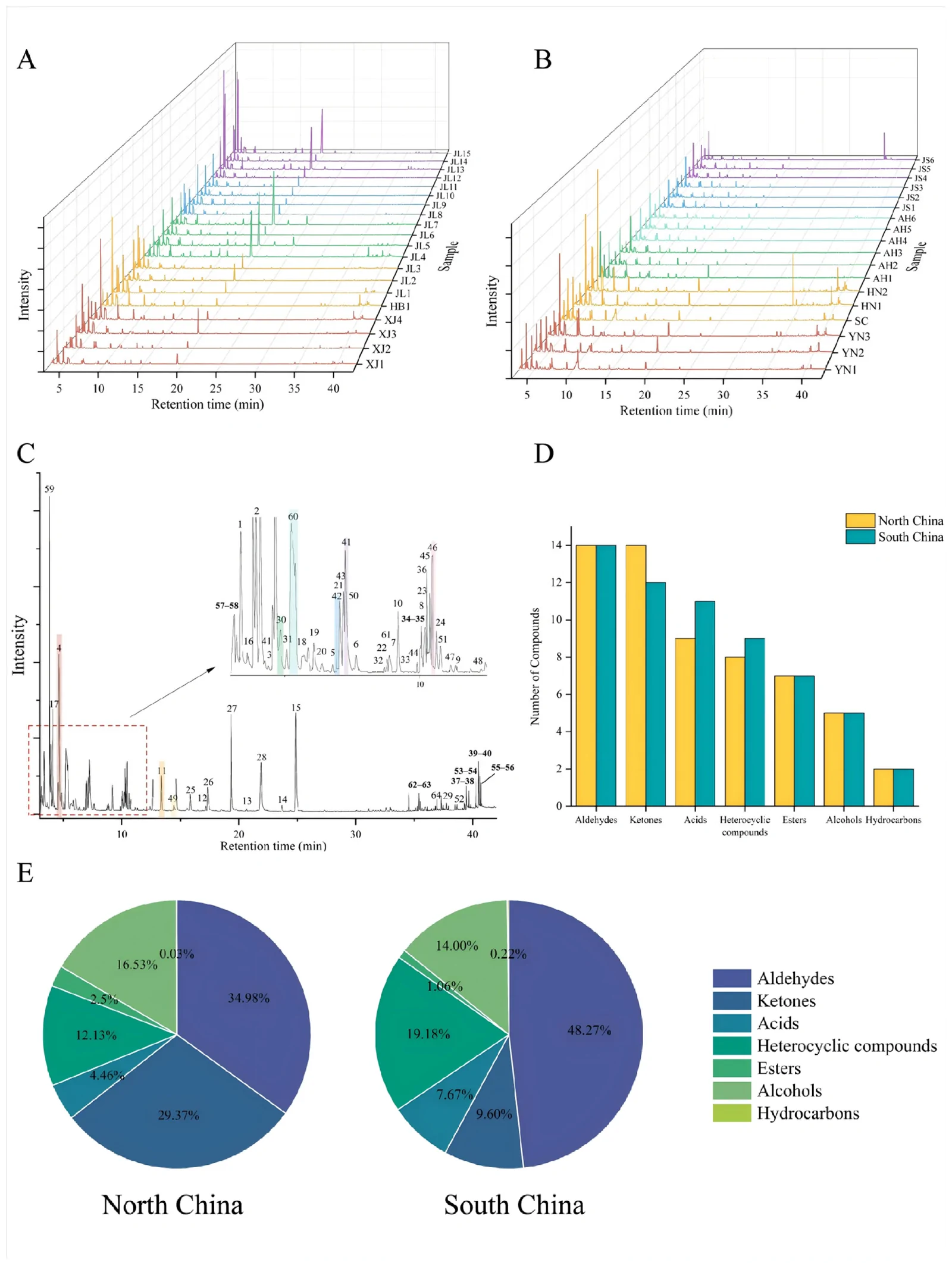
Analysis results of volatile components in chicory from North and South China. (**A**) TICs of chicory from North China. (**B**) TICs of chicory from South China. (**C**) TICs of 64 volatile components (Peaks 4, 11, 30, 41, 42, 46, and 60 are common to all origins). (**D**) Types of volatile components from South and North China. (**E**) Relative contents of volatile components from North and South China.

Under the above conditions, volatile components in chicory were detected, with the total ion current chromatograms (TICs) shown in Figure 1A,B. Combined with NIST14 database searching, 64 volatile components (similarity > 85) were identified in chicory samples from different origins, including 15 aldehydes, 14 ketones, 11 acids, 9 heterocyclics, 7 esters, 5 alcohols, and 3 hydrocarbons (Table S1, Figure 1C). Peak-area normalization analysis revealed that aldehydes accounted for 40.29% of the total peak area, being the main volatile components; this is followed by ketones (22.85%) and heterocyclic compounds (15.52%), which is consistent with the previous findings [26]. The grassy odor of aldehydes may contribute to the faint smell of raw chicory, while heterocyclic compounds (mainly pyrazines and furans) impart a roasted aroma and caramel-like sweetness, which also explains the unique sensory characteristics of chicory as a coffee substitute [27]. Notably, Furfural (22.93%), 2,3-Dihydro-3,5-dihydroxy-6-methyl-4H-pyran-4-one (14.93%), and Furfuryl alcohol (9.90%) were the three most abundant components, all exhibiting significant antioxidant activity [19,28].

For samples from southern regions, 61 volatile components were identified; compared with northern regions, there were 2 more species in acids, heterocyclics, and hydrocarbons, respectively, and 2 fewer in ketones (Figure 1D). Peaks 4, 11, 30, 41, 42, 46, 49 and 60 are common to all origins and marked by different color. The types and relative contents of volatile compounds in chicory from different sources are shown in Figure 1E. A total of 59 volatile components were identified in samples from northern regions, including 16 aldehydes, 14 ketones, 9 acids, 8 heterocyclics, 7 esters, 5 alcohols, and 2 hydrocarbons. The types and quantity structure of volatile components in samples from the two regions were generally similar, but significant differences in contents were observed, which may be related to multiple ecological factors such as climate, light, altitude, soil composition, and water content [29,30].

#### 2.1.2. Chemometric Analysis

**Figure 2:**
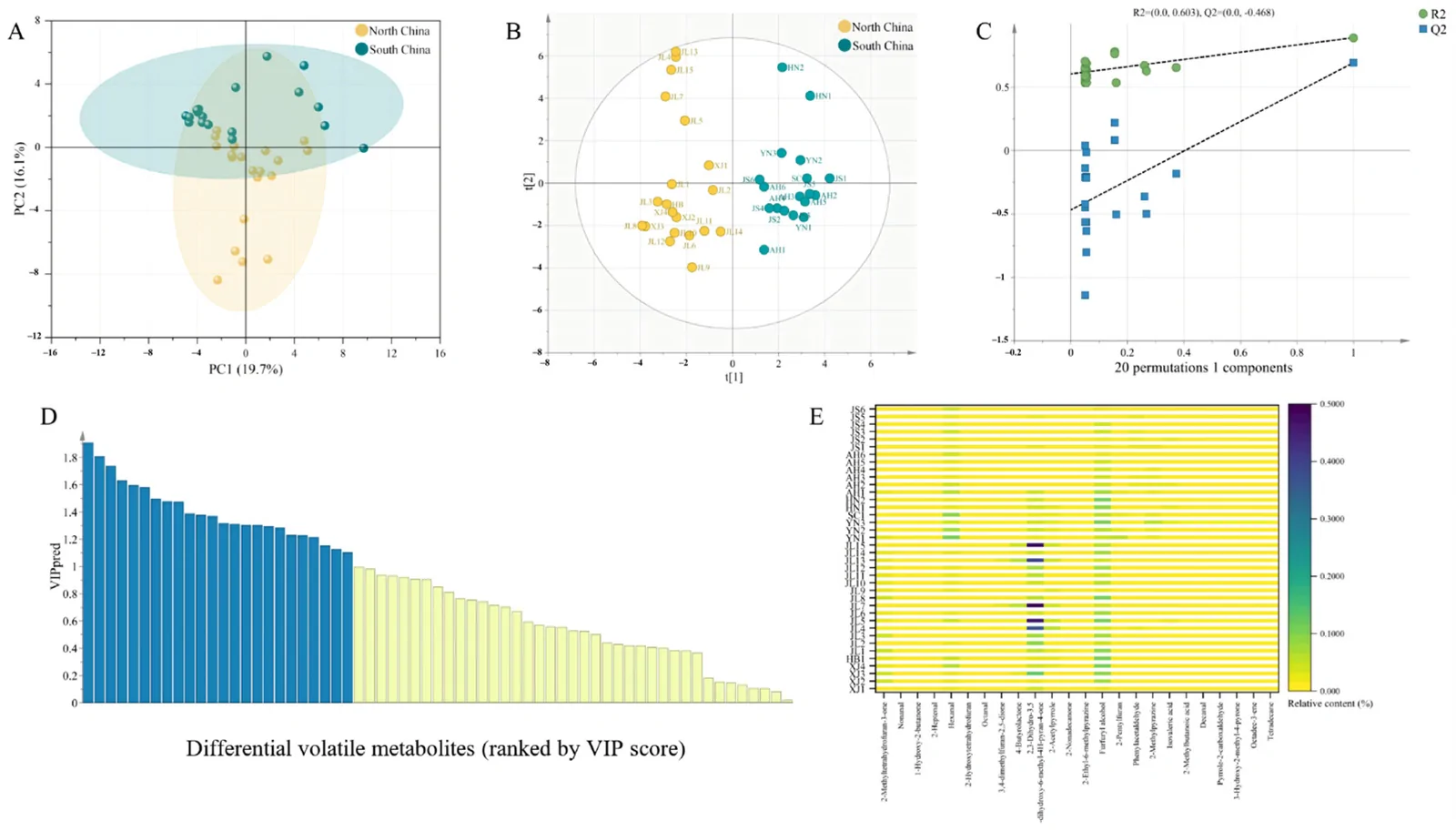
Chemometric analysis of volatile components in chicory from North and South China. (**A**) Principal Component Analysis (PCA) score scatter plot (The yellow ellipse is the 95 % confidence region of chicory samples from northern China, and the blue ellipse is the 95 % confidence region of chicory samples from southern China). (**B**) Orthogonal Partial Least Squares-Discriminant Analysis (OPLS-DA) score scatter plot. (**C**) Permutation test plot (The regression line corresponding to the imaginary line (green) R2: the regression fitting line of R2; the regression line corresponding to the imaginary line (blue) Q2: The regression fitting line of Q2). (**D**) Variable Importance in Projection (VIP) score plot (Blue represents the component with VIP value > 1, and yellow represents the component with VIP value < 1). (**E**) Heatmap analysis.

Principal Component Analysis (PCA) is an unsupervised analytical method for data dimensionality reduction, where information in the original data is represented by the extracted principal components (PCs). As shown in the PCA score plot, the contribution rates of the first principal component (PC1) and the second principal component (PC2) were 19.7% and 16.1%, respectively (Figure 2A). All 38 sample batches fell within the confidence interval; however, the intervals of samples from northern and southern regions overlapped with each other, showing a tendency toward separation but without distinct clustering. To further distinguish the effects of different regions on the volatile components in chicory and screen key metabolites, OPLS-DA was employed. OPLS-DA is a supervised discriminant analysis method that can maximize intergroup differences while minimizing intragroup differences [31]. The response values of 64 volatile metabolites from 38 batches of chicory were imported into SIMCA 14.1 for OPLS-DA analysis. Herein, R^2^X and R^2^Y represent the explanatory rates of the model for X and Y matrices, respectively, and Q^2^ denotes the predictive ability of the model. R^2^Y and Q^2^ values > 0.5 indicate acceptable model fitting, with values closer to 1 reflecting better model performance [32]. The analysis results showed R^2^Y = 0.889 and Q^2^ = 0.692, indicating that the model had strong explanatory power for data variation and reliable predictive efficiency. Chicory samples from different geographical origins showed clear separation in the score plot (Figure 2B). A permutation test with 200 iterations was performed to verify potential overfitting of the model, yielding R^2^ and Q^2^ intercepts of 0.607 and −0.582, respectively, with R^2^ > 0 and Q^2^ < 0.05, confirming the good reliability and validity of the model (Figure 2C).

Variable Importance in Projection (VIP) was used to screen differential markers among different origins. Generally, variables with VIP values exceeding 1 are considered to play important roles [33]. The VIP plot showed that 24 volatile metabolites made significant contributions to origin classification (VIP > 1), ranked by contribution from high to low as follows: 2-Methyltetrahydrofuran-3-one, Nonanal, 1-Hydroxy-2-butanone, 2-Heptenal, Hexanal, 2-Hydroxytetrahydrofuran, Octanal, 3,4-dimethylfuran-2,5-dione, 4-Butyrolactone, 2,3-Dihydro-3,5-dihydroxy-6-methyl-4H-pyran-4-one, 2-Acetylpyrrole, 2-Nonadecanone, 2-Ethyl-6-methylpyrazine, Furfuryl alcohol, 2-Pentylfuran, Phenylacetaldehyde, 2-Methylpyrazine, Isovaleric acid, 2-Methylbutanoic acid, Decanal, Pyrrole-2-carboxaldehyde, 3-Hydroxy-2-methyl-4-pyrone, Octadec-3-ene, and Tetradecane (Figure 2D). These 24 compounds can serve as potential volatile markers for distinguishing chicory from southern and northern origins.

Further heatmap analysis of these 24 differential volatile metabolites is shown in Figure 2E. Darker colors indicate higher relative contents. The results showed that for 2,3-Dihydro-3,5-dihydroxy-6-methyl-4H-pyran-4-one, the northern chicory samples exhibited darker colors in the heatmap, with an average relative content of 0.28% (southern chicory: 0.10%). Similarly, Furfuryl alcohol also presented darker colors in northern chicory samples, corresponding to an average relative content of 0.21% in the north and 0.11% in the southern samples. By contrast, Hexanal showed darker colors in southern chicory samples, and its average relative content was 0.09% (northern chicory: 0.03%). Therefore, these key volatile metabolites may affect the antioxidant efficacy of chicory from different sources, and in-depth analysis of these 24 key biomarkers will be conducted in subsequent studies.

### 2.2. UPLC-Q-TOF-MS Analysis of Chicory from Different Regions

#### 2.2.1. Identification of Non-Volatile Metabolites

**Figure 3:**
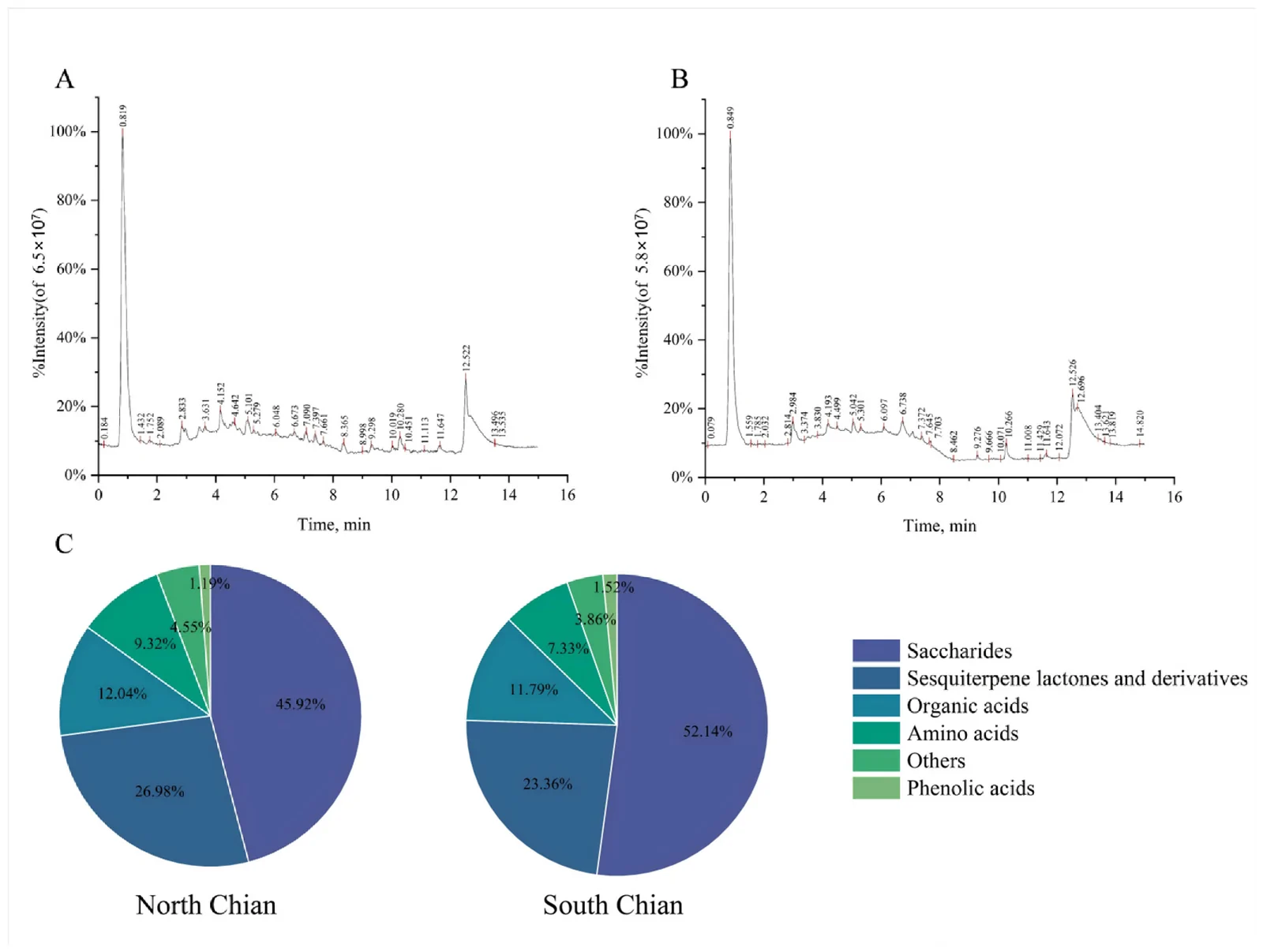
Determination results of non-volatile components in chicory from North and South China. (**A**) Positive-mode TICs. (**B**) Negative-mode TICs. (**C**) Relative content of non-volatile components from North and South China.

TICs of chicory from different origins were obtained under positive and negative ion modes, as shown in Figure 3A,B. Components with secondary fragment ions were identified using Peakview 1.2 and MS Dial ver.5.1 software, combined with self-built databases, public databases, reference standards for the five HPLC-quantified phenolic acids, and relevant literature. A total of 35 components were identified from chicory of different origins, including 16 sesquiterpene lactones and their derivatives, 5 saccharides, 4 amino acids, 4 organic acids, 3 phenolic acids, 1 fatty acid, 1 alkaloid, and 1 vitamin.

Relative contents of non-volatile components in chicory from different sources are shown in Figure 3C. A total of 35 non-volatile components were identified in both southern and northern samples, but differences in the contents of non-volatile metabolites were observed between chicory from different origins, requiring further analysis.

#### 2.2.2. Identification of Sesquiterpene Lactones and Their Derivatives

A total of 16 sesquiterpene lactones and their derivatives were identified in chicory (1–16). For example, the precursor ion with *m*/*z* 277.1073 [M + H]^+^ was posited to possess the molecular formula of C_15_H_16_O_5_. The principal secondary fragment ions were *m*/*z* 258.8943 [M − H_2_O + H]^+^, *m*/*z* 213.0909 [M − H_2_O − CO_2_ + H]^+^, and *m*/*z* 185.0954 [M - H_2_O-CO_2_-CO_2_H_4_ + H]^+^, determined through public database comparisons and relevant literature, confirming that it was identified as Lactucin. The cleavage pattern is illustrated in Figure S1A. Furthermore, additional structurally similar compounds 1, 4, 7, 8, 9, 11, 12, 13, 14, 15 and 16 were identified based on this cleavage pattern and related literature [34,35]. Based on the parent ion *m*/*z* 461.1414 [M + Na]^+^ and the major secondary fragment ions *m*/*z* 443.1286 [M-H_2_O + Na]^+^, *m*/*z* 299.0625 [M-H_2_O-C_6_H_8_O_4_ + Na]^+^, and *m*/*z* 281.0519 [M-2H_2_O-C_6_H_8_O_4_ + Na]^+^, the potential molecular formula C_21_H_26_O_10_ for Cichorioside K was postulated through a comparison with public databases. Figure S1B illustrates the cleavage pathway. The molecular formula of the parent ion *m*/*z* 593.2181 [M + Na]^+^ was hypothesized to be C_27_H_38_O_13_, and its major secondary fragmentation ions were *m*/*z* 447.1606 [M-H_2_O-C_6_H_8_O_3_ + Na]^+^, followed by further fragmentation into *m*/*z* 285.1077 [M-H_2_O-C_6_H_8_O_3_-C_6_H_10_O_5_ + Na]^+^ and *m*/*z* 266.5968 [M-2H_2_O-C_6_H_8_O_3_-C_6_H_10_O_5_ + Na]^+^. According to the public database comparison, it was identified as Cichorioside D. The cleavage pathway is shown in Figure S1C.

#### 2.2.3. Identification of Saccharides

A total of 5 saccharides (17–21) were determined in chicory. The presumed molecular formula of the parent ion *m*/*z* 683.2533 [M − H]^−^ was C_24_H_44_O_22_, with its principal secondary fragment ion at *m*/*z* 341.1068 [M-C_12_H_22_O_11_-H]^−^, which was further fragmented into *m*/*z* 179.0558 [M-C_12_H_22_O_11_-C_6_H_10_O_5_-H]^−^ and *m*/*z* 161.0451 [M-C_12_H_22_O_11_-C_6_H_12_O_6_-H]^−^. It was verified as Stachyose through comparisons with public databases, and the cleavage process is shown in Figure S1D.

#### 2.2.4. Identification of Amino Acid Compounds

Four amino acids (22–25) were detected in chicory. The chemical formula of the parent ion *m*/*z* 175.1190 [M + H]^+^ was proposed as C_6_H_14_N_4_O_2_, with a predominant secondary fragmentation ion of *m*/*z* 130.0984 [M-CHO_2_ + H]^+^, followed by a further fragmentation into *m*/*z* 70.0647 [M-CHO_2_-CH_6_N_3_ + H]^+^ and *m*/*z* 60.0555 [M-CHO_2_-C_4_H_8_N + H]^+^. It was identified as L-Arginine based on public database comparisons and related literature, with the cleavage pathway represented in Figure S1E.

#### 2.2.5. Identification of Phenolic Acid Compounds

A total of 4 organic acids were identified in chicory (26–29). The molecular formula of the precursor ion *m*/*z* 329.2334 [M − H]^−^ was determined to be C_18_H_34_O_5_, with its principal secondary fragment ions being *m*/*z* 229.1451 [M-C_6_H_10_-H_2_O-H]^−^ and *m*/*z* 211.1346 [M-C_6_H_10_-2H_2_O-H]^−^. It was identified as Pinellic acid based on public database comparisons and relevant literature reports [36], and the cleavage pattern is shown in Figure S1F. Furthermore, other compounds were identified based on secondary fragments and literature reports.

#### 2.2.6. Identification of Organic Acid Compounds

Three phenolic acids were determined in chicory (30–32). The molecular formula of the precursor ion *m*/*z* 353.0869 [M − H]^−^ was assigned as C_16_H_18_O_9_, while the secondary fragment ions were found as *m*/*z* 191.055 [M-C_9_H_6_O_3_-H]^−^ and *m*/*z* 179.034 [M-C_7_H_10_O_5_-H]^−^. Based on a comparison with a reference substance and a relevant literature report [37], it was identified as Chlorogenic acid.

#### 2.2.7. Identification of Other Compounds

In addition, 3 other components (33–35) were detected in chicory. For example, the presumed molecular formula of the parent ion *m*/*z* 217.0972 [M + H]^+^ is C_12_H_12_N_2_O_2_, and its major secondary fragment ions were *m*/*z* 173.1071 [M − CO_2_ + H]^+^, *m*/*z* 144.0816 [M-CO_2_-CH_3_N + H]^+^, and *m*/*z* 118.0371 [M-CO_2_-CH_3_N-C_2_H_2_ + H]^+^. This compound had been identified as 2,3,4,9-Tetrahydro-1H-beta-carboline-3-carboxylic acid through literature and database comparisons, with the cleavage process depicted in Figure S1G. Additionally, other components were verified by analyzing secondary fragment ions, comparing with public databases, and referring to the literature [38].

#### 2.2.8. Chemometric Analysis

**Figure 4:**
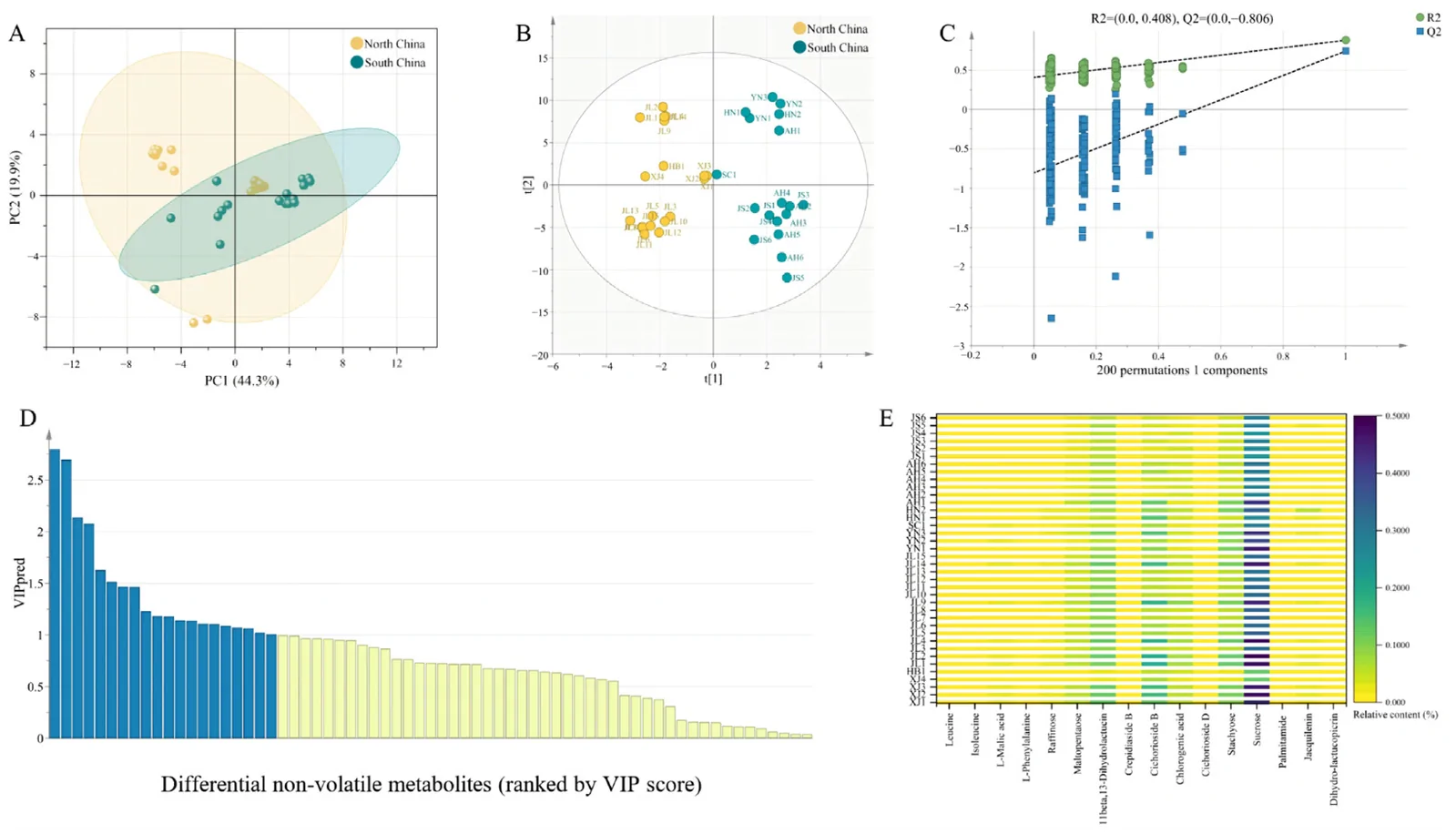
Chemometric analysis of non-volatile components in chicory from North and South China. (**A**) PCA score scatter plot (The yellow ellipse is the 95 % confidence region of chicory samples from northern China, and the blue ellipse is the 95 % confidence region of chicory samples from southern China). (**B**) OPLS-DA score scatter plot. (**C**) Permutation test plot (The regression line corresponding to the imaginary line (green) R2: the regression fitting line of R2; the regression line corresponding to the imaginary line (blue) Q2: The regression fitting line of Q2). (**D**) VIP score plot (Blue represents the component with VIP value > 1, and yellow represents the component with VIP value < 1). (**E**) Heatmap analysis.

PCA was performed on the non-volatile components of 38 sample batches of chicory. As shown in the score scatter plot, the first principal component (PC1) and the second principal component (PC2) accounted for 44.3% and 19.9% of the total variance, respectively (Figure 4A). It can be observed that under unsupervised analysis, the chicory samples from northern and southern regions showed partial overlap and failed to achieve effective separation. To screen potential markers for quality differences in chicory and compare their geographical variations, data were imported into SIMCA 14.1 software for chemometric analysis. OPLS-DA was used for geographical origin differentiation, and the results showed that the model had R^2^Y = 0.878 and Q^2^ = 0.740, indicating good reliability and strong predictive ability. The OPLS-DA score scatter plot showed that chicory could be clearly divided into two groups based on geographical origin (Figure 4B). A permutation test with 200 iterations was performed to evaluate the validity and predictability of the model, yielding model parameters R^2^ = 0.408 and Q^2^ = −0.806, with R^2^ > 0 and Q^2^ < 0.05, confirming no overfitting (Figure 4C).

VIP was used to screen significantly differential components in chicory from different origins, and a total of 16 components with VIP > 1 were identified (Figure 4D), ranked by contribution from high to low as follows: Leucine, Isoleucine, L-Malic acid, L-Phenylalanine, Raffinose, Maltopentaose, 11β,13-Dihydrolactucin, Crepidiaside B, Cichorioside B, Chlorogenic acid, Cichorioside D, Stachyose, Sucrose, Palmitamide, Jacquilenin, and Dihydro-lactucopicrin.

Further heatmap analysis of these 16 differential non-volatile components (Figure 4E) showed that for Cichorioside B, the northern chicory samples exhibited darker colors in the heatmap, with an average relative content of 0.18% (southern chicory: 0.13%); similarly, Maltopentaose also presented darker colors in northern chicory samples, with a corresponding average relative content of 0.11% (southern chicory: 0.07%); in addition, 11β,13-Dihydrolactucin exhibited a distribution pattern consistent with the above two compounds—the northern chicory samples showed darker colors in the heatmap, and their average relative content was 0.12% (southern chicory: 0.08%). Environmental factors such as soil nutrients, light intensity, and temperature gradients may affect the synthesis and accumulation of sesquiterpene lactones by regulating secondary metabolic pathways, leading to differences in the contents of these compounds in chicory from different origins [39,40]. These compositional differences may result in variations in antioxidant activity between southern and northern chicory, and their specific mechanisms need to be further verified by in vitro experiments and molecular docking techniques.

### 2.3. Machine Learning Analysis

#### 2.3.1. Differentiation of Chicory Producing Areas Based on Machine Learning

**Figure 5:**
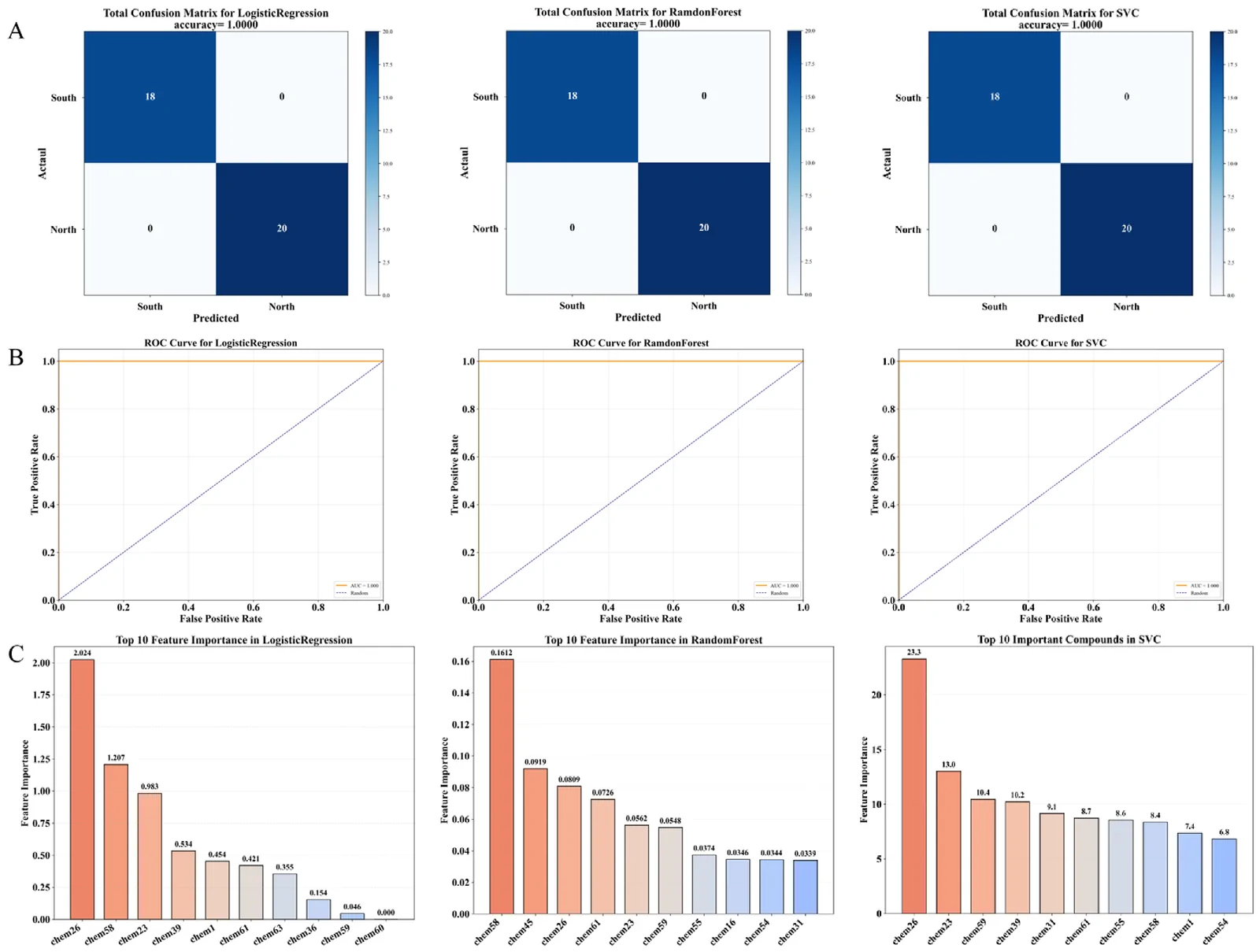
Volatile chemical markers prediction in chicory from North and South China. (**A**) Three machine learning confusion matrices (LR, RF, SVC from left to right). (**B**) ROC curve. (**C**) Feature importance bar chart.

**Figure 6:**
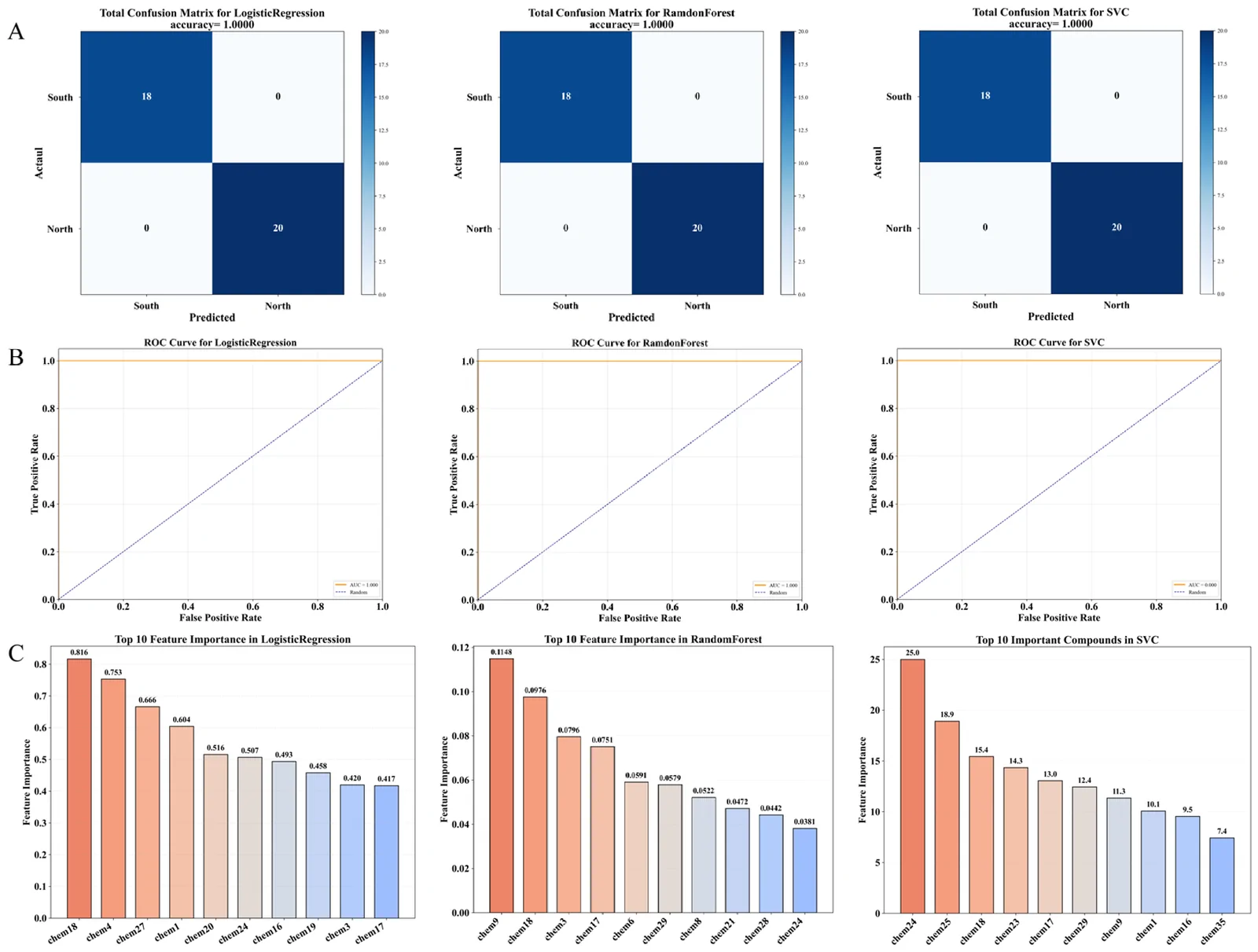
Non-volatile chemical markers prediction in chicory from North and South China. (**A**) Three machine learning confusion matrices (LR, RF, SVC from left to right). (**B**) ROC curve. (**C**) Feature importance bar chart.

Preliminary classification of the volatile and non-volatile components from 38 sample batches of chicory was performed using chemometric methods. Compared with PCA and OPLS-DA methods, machine learning exhibits superior capabilities in data processing and classification model construction. To achieve better classification and prediction of chemical biomarkers for chicory samples from different producing regions, three machine learning models were established for further analysis: Logistic Regression, Random Forest, and Support Vector Classification. Data from GC-MS and UPLC-Q-TOF/MS were separately organized into two M × N data matrices, where M corresponds to the number of sample batches and N corresponds to the number of identified compounds. To eliminate differences in dimensionality and value ranges among features, the data matrices were subjected to Z-score standardization, missing values were imputed with 0, and the dataset was randomly split into a training set and a test set at a ratio of 0.7:0.3. After model training, confusion matrices were generated for all samples to evaluate the classification performance of the models. In the confusion matrix, correctly predicted results are arranged along the diagonal, while misclassified results are scattered on both sides of the diagonal. As shown in Figure 5A and Figure 6A, all three ML models could accurately distinguish samples from northern and southern regions based on their confusion matrices, with the classification accuracy of each model being 1.0. Meanwhile, the receiver operating characteristic (ROC) curves of all models closely approached the upper left corner. The closer the area under the ROC curve is to 1, the more reliable the model, indicating that the three models all exhibit accurate recognition performance for chicory from different producing regions (Figure 5B and Figure 6B).

In the original analysis, the three models achieved a classification accuracy of 1.0 under a single 7:3 training/test split. To obtain a more realistic estimate of generalization performance and to address the possibility of overfitting, repeated stratified 5-fold cross-validation (5 folds × 20 repeats) was further performed. As shown in Table 1, the mean accuracy ranged from 0.882 to 0.939 for the volatile dataset and from 0.873 to 0.937 for the non-volatile dataset, with mean AUC values of 0.961–0.993 and 0.893–0.989, respectively. The standard deviations of accuracy (0.077–0.124) and AUC (0.034–0.142) were within the range expected for small-sample cross-validation. These results indicate that the accuracy obtained from the single training/test split was somewhat optimistic, but that the models still achieved reproducible discrimination between northern and southern samples, with all mean accuracy values above 0.87 and all mean AUC values above 0.89.

#### 2.3.2. Identification of Chemically Discriminative Compounds in Chicory Based on Machine Learning

Subsequently, feature importance analysis was performed on the three trained machine learning models to identify the features and their corresponding weights used for classification. Feature importance bar plots revealed the top 10 screened compounds under different machine learning models (Figure 5C and Figure 6C). It can be observed that the contribution order of biomarkers generated by different models varies. This may be attributed to the distinct processing strategies of different models for feature extraction, feature selection, and model architecture, which consequently influence the evaluation of feature variable importance. The three algorithms are statistically independent; thus, the intersection of relevant features was extracted, yielding 8 volatile and 7 non-volatile potential chemical biomarkers, respectively. The identified compounds are as follows: 8 volatile compounds included 2-Nonadecanone, 2-Methyltetrahydrofuran-3-one, 2,3-Dihydro-3,5-dihydroxy-6-methyl-4H-pyran-4-one, Octadec-3-ene, Tetradecane, 2-Pentylfuran, 2-Acetylpyrrole, and 2-Methylpyrazine; 7 non-volatile compounds included Lactucin 15-oxalate, Cichorioside B, Chlorogenic acid, Raffinose, Maltopentaose, Leucine, and L-Malic acid.

Machine learning enabled the effective differentiation of northern and southern chicory samples with high model accuracy, outperforming the classification efficacy of PCA and the predictive capacity of OPLS-DA (Q^2^ = 0.692–0.740). This demonstrates the excellent capability of machine learning in classifying chicory samples from different producing regions, thereby ensuring the reliability of chemical biomarker identification. On the basis of accurate classification, the chemical features screened by each model varied distinctly, further highlighting the importance of employing multiple analytical approaches for chemical biomarker prediction. In order to comprehensively investigate the compounds that cause the difference between the northern and southern chicory, the results of chemometric analysis were combined with machine learning. The compounds predicted by machine learning were largely included in the results of chemometric analysis, indicating that the improvement in model accuracy enables the further validation of chemical biomarkers for chicory from different producing regions. It should be noted that there is an imbalance in geographic sampling. However, the results shown significant overlap between the discriminatory metabolites identified through chemometrics (OPLS-DA VIP > 1) and those screened using three machine learning models (LR, RF, and SVC): most of the compounds predicted by machine learning were included in the chemometric analysis results, and key volatile and non-volatile biomarkers were consistently identified across the different analytical methods. The consistency observed between these two methodologically independent approaches indicates that the north–south metabolic differences observed in this study are stable.

### 2.4. Quantitative Analysis of Bioactive Ingredients in Chicory

#### 2.4.1. Methodological Validation

The blank, mixed control and sample solutions were analyzed according to the chromatographic conditions, as shown in Figure S2A. The separations of Chlorogenic acid, Chicoric acid, Isochlorogenic acid B, Isochlorogenic acid A and Isochlorogenic acid C from the neighboring peaks were all greater than 1.5, with the range of tailing factor being 0.95~1.10, and a theoretical plate count exceeding 2000, indicating the method’s robust system adaptability.

We accurately measured 0.10, 0.50, 1.00, 2.00, 4.00, 8.00, 10.00 mL of the mixed control solution, before transferring it into a 10 mL volumetric flask and adding methanol to prepare the series of mixed control solutions. Subsequently, sample solution was injected for determination. The concentration x (μg·mL^−1^) and peak area y of each control solution were used as the horizontal and vertical coordinates, respectively, to fit the standard curve. The mixed control solution was sequentially diluted and the limits of quantification and detection were calculated according to the signal-to-noise ratios of 10:1 and 3:1, respectively, as presented in Table S2.

The mixed control solution was measured continuously for 6 times, with the RSDs of the peak areas of Chlorogenic acid, Chicoric acid, Isochlorogenic acid B, Isochlorogenic acid A and Isochlorogenic acid C being 0.54%, 0.52%, 0.55%, 0.78% and 0.57%, respectively, indicating that the instrument exhibited commendable precision. The sample solution was prepared by taking AH1 chicory powder, placed at room temperature for 0, 2, 4, 8, 12 and 24 h, and then measured, with the peak area RSDs of Chorizonic acid, Chicoric acid, Isochorizonic acid B, Isochorizonic acid A and Isochorizonic acid C being 1.0%, 1.0% and 1.0%, respectively. The RSDs of the peak areas of Chlorogenic acid, Chicoric acid, Isochlorogenic acid B, Isochlorogenic acid A and Isochlorogenic acid C were 1.37%, 1.30%, 1.37%, 1.71% and 1.74%, respectively, demonstrating that the sample solutions remained stable for 24 h. Six sample solutions were prepared by taking the powder of AH1 sample and determined according to the HPLC conditions. The RSDs of the peak areas of Chlorogenic acid, Chicoric acid, Isochlorogenic acid B, Isochlorogenic acid A and Isochlorogenic acid C were 1.03%, 1.50%, 1.19%, 0.95% and 1.45%, respectively, showing the method reproducibility.

AH1 chicory powder (0.5 g) with a known concentration (*n* = 6) was accurately measured, and Chlorogenic acid, Chicoric acid, Isochlorogenic acid B, Isochlorogenic acid A and Isochlorogenic acid C were added in appropriate quantities, approximately equating to 100% of the sample’s content. Sample solutions were prepared and measured according to the HPLC conditions. The mean recoveries for the peak concentrations of Chlorogenic acid, Chicoric acid, Isochlorogenic acid B, Isochlorogenic acid A and Isochlorogenic acid C were 100.4%, 101.7%, 98.9%, 103.2% and 101.3%, with RSDs of 1.47%, 1.25%, 1.59%, 1.95% and 1.77%, suggesting commendable method accuracy.

#### 2.4.2. Content Determination

We accurately weighed the powder from each batch of chicory samples, prepared the test solution, and performed the determination according to the HPLC conditions. The peak areas of the chromatographic peaks were documented, and the concentrations of the five components were determined based on the established standard curves. The findings indicated that the concentrations of Chlorogenic acid, Chicoric acid, Iochlorogenic acid B, Isochlorogenic acid A and Isochlorogenic acid C ranged from 5.120 to 57.61 μg·g^−1^, 0.4228 to 5.257 μg·g^−1^, 1.871 to 32.20 μg·g^−1^, 2.922 to 30.74 μg·g^−1^ and 0.4399 to 5.787 μg·g^−1^, respectively. The content of Chlorogenic acid in northern chicory was significantly higher than that in southern chicory (*p* < 0.05); the content of Chicoric acid was comparatively low in northern chicory; the content of Isochlorogenic acid B was relatively high in southern chicory; the difference in the content of Isochlorogenic acid A between northern and southern chicory was relatively minimal; and the content of Isochlorogenic acid C was relatively low in both northern and southern chicory, with no significant difference observed (Figure S2B). Considering that phenolic acids function as natural antioxidants [41,42], these findings reveal significant disparities in the concentrations of these compounds in chicory from the northern and southern regions. This not only offers empirical support for further exploration of the quality characteristics and underlying causes of these regional differences but also implies that variations in component concentrations may result in divergent bioactivities between the northern and southern chicory. Consequently, it is sensible to conduct subsequent studies to further clarify the intrinsic correlation.

### 2.5. Results of Network Pharmacology Prediction

#### 2.5.1. Prediction of Active Metabolites and Potential Targets

A total of 99 compounds were identified by HS-GC-MS and UPLC-Q-TOF-MS, and 13 antioxidant metabolites were screened according to the criteria of OB ≥ 30 and DL ≥ 0.18. Using the SwissTargetPrediction database, 355 potential targets of these 13 antioxidant metabolites were identified. To explore the interaction between antioxidant metabolites and antioxidant targets, a “chicory–metabolite–target” network diagram was constructed (Figure S3A). This network contains 369 nodes (1 chicory node, 13 antioxidant metabolite nodes, and 355 antioxidant target nodes) and 661 edges. More edges between nodes indicate more significant interactions. The larger the circular area of a node in the diagram, the higher its degree value. The top 3 active components by degree value were Lactucopicrin, Cichorioside B, and 11beta,13-Dihydro-8-deoxylactucin, which may be the key metabolites for antioxidant stress in chicory. The results showed that antioxidant metabolites can act on multiple targets, and different antioxidant metabolites can jointly act on the same target, reflecting the synergistic characteristics of multi-component and multi-target effects of traditional Chinese medicine [43].

In addition, 1025 antioxidant-related targets (with a score > 10) were retrieved from the Genecards and DrugBank databases. A total of 91 intersection targets between antioxidant metabolites and antioxidant targets were identified by Venn analysis (Figure S3B). To screen the core antioxidant targets, the 91 intersection targets were uploaded to the STRING database with the minimum interaction confidence threshold set to >0.4. A PPI network was constructed and visualized using Cytoscape 3.8.0 (Figure S3C), where the size and color depth of targets are determined by their degree values. The results yielded 89 nodes and 958 edges, and the DC, CC, and BC of network nodes were calculated. First, 23 targets were screened out with all three indices greater than the mean values (DC > 21.5281, CC > 0.0062, BC > 76.7640). Then, 7 core antioxidant targets were identified by degree value ranking: Interleukin-6 (IL6, PDB ID:5SFK), recombinant signal transducer and activator of transcription 3 (STAT3, PDB ID:6TLC), peroxisome proliferator activated receptor gamma (PPARG, PDB ID:8B8X), Jun proto-oncogene (JUN, PDB ID:8SOS), epidermal growth factor receptor (EGFR, PDB ID:3P0Z), mitogen-activated protein kinase 3 (MAPK3, PDB ID:4QTB), and B-cell lymphoma 2 (BCL2, PDB ID:9O16).

#### 2.5.2. GO and KEGG Analysis

To clarify the biological functions of target genes and their involvement in various signaling pathways, GO functional enrichment analysis and KEGG pathway enrichment analysis were performed on the intersection targets [22]. With *p* < 0.05 as the screening criterion, GO enrichment analysis yielded 1845 BP, 56 CC, and 183 MF; KEGG enrichment analysis identified 242 pathways. The top 5 pathways for each category were visualized (Figure S3D). A larger value on the abscissa (−log10 (*p* value)) indicates more significant functional enrichment; a larger number of targets (count) indicates more targets related to the function.

GO enrichment results showed that BP mainly involved positive regulation of phosphorus metabolic process, positive regulation of phosphate metabolic process, response to peptide hormone, response to exogenous substance stimulus, and positive regulation of the MAPK cascade; CC mainly involved membrane raft, membrane microdomain, distal axon, nuclear envelope, and vesicle lumen; MF mainly involved histone H3 kinase activity, histone kinase activity, histone H2AX kinase activity, protein serine/threonine kinase activity, and protein serine kinase activity.

KEGG enrichment results showed that the top 5 signaling pathways were classified into three categories: human diseases (2 pathways), biological systems (2 pathways), and environmental information processing (1 pathway), including lipid and atherosclerosis, the gonadotropin-releasing hormone (GnRH) signaling pathway, the C-type lectin receptor signaling pathway, insulin resistance, and the vascular endothelial growth factor (VEGF) signaling pathway, suggesting that chicory may exert antioxidant effects through these pathways. As a key neurohormone, GnRH regulates the hypothalamic–pituitary–gonadal (HPG) axis, a core pathway for reproductive function regulation. Recent studies have found that GnRH and its receptors are expressed in extragonadal tissues such as the heart, blood vessels, and immune cells, indicating that their physiological roles extend to cardiovascular health [44]. C-type lectin receptors (CLRs) are present in myeloid cells, and ligand binding to CLRs can coordinate immune signal generation [45]. These results indicate that the antioxidant effect of chicory may involve the regulation of cardiovascular diseases, metabolic diseases, and immune-related diseases, and it has potential application value in the prevention and treatment of these diseases.

### 2.6. Molecular Docking Between Key Antioxidant Metabolites and Core Antioxidant Targets

To characterize the interaction between key antioxidant metabolites and core regulatory proteins, molecular docking was used to verify the interactions between 3 key antioxidant metabolites with high degree values (Lactucopicrin, Cichorioside B, 11β,13-Dihydro-8-deoxylactucin) and 7 core antioxidant targets with high degree values (IL6, STAT3, PPARG, JUN, EGFR, MAPK3, BCL2) (figure omitted). The lower the binding free energy, the more stable the binding between ligand and receptor: a binding energy < 0 kcal·mol^−1^ indicates spontaneous binding, <−1.2 kcal·mol^−1^ indicates good binding activity, and <−5.0 kcal·mol^−1^ indicates strong binding affinity. As shown in Figure S4A, the binding energies of all compounds were <−1.2 kcal·mol^−1^, indicating that the 3 antioxidant metabolites have good affinity with the selected targets, verifying the validity of the metabolites and core targets identified in this study.

The three best docking results were visualized using PyMOL: Cichorioside B had the highest binding affinity with BCL2 (−9.9 kcal·mol^−1^) (Figure S4B) and formed 9 hydrogen bonds with ARG-87, ARG-42, ILE-80, GLN-45, GLU-46, and GLU-50 of BCL2. Generally, more hydrogen bonds indicate more stable binding and lower energy scores [44]. Lactucopicrin had the second highest binding affinity with STAT3 (−9.3 kcal·mol^−1^) (Figure S4C), forming 6 hydrogen bonds with SER-59, SER-540, ASN, TYR-539, and TRP-501 of STAT3, and hydrophobic interactions with GLU-506 and LEU-525. Cichorioside B had the third highest binding affinity with EGFR (−9.2 kcal·mol^−1^) (Figure S4D), forming 6 hydrogen bonds with GLN-812, ASN-816, ASP-974, and GLN-976 of EGFR, and hydrophobic interactions with ARG-973 and ASP-974.

### 2.7. Molecular Dynamics Simulation

Molecular dynamics simulation can analyze the dynamic changes and mechanisms of protein conformation, observe ligand–protein complexes in dynamic systems, and reveal conformational flexibility and motion characteristics that cannot be reflected by static structures [46]. Based on the molecular docking results, 100 ns molecular dynamics simulations were performed on BCL2-Cichorioside B and EGFR-Cichorioside B complexes to analyze their dynamic characteristics.

**Figure 7:**
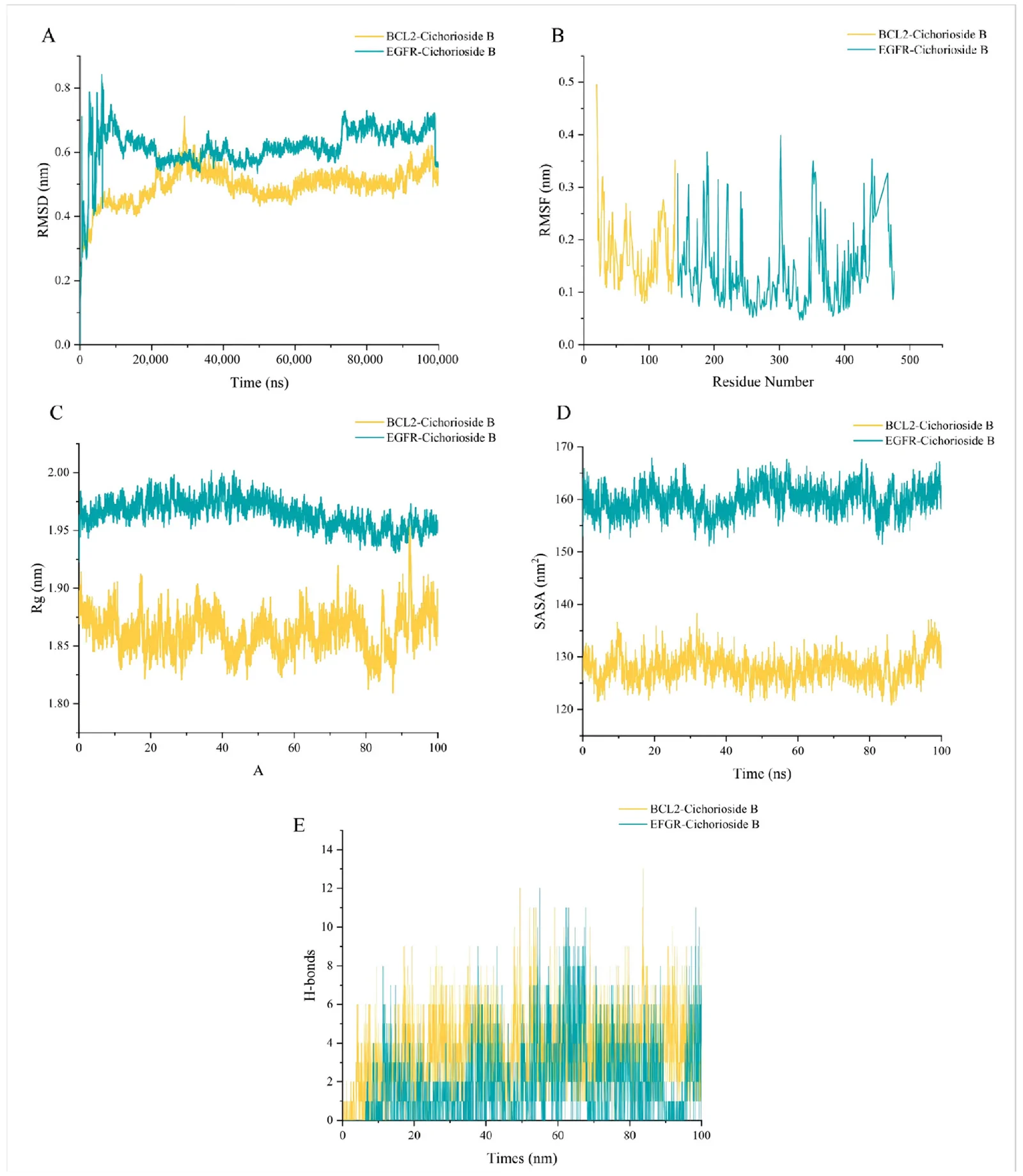
Results of molecular dynamics simulation. (**A**) RMSD. (**B**) RMSF. (**C**) Rg. (**D**) SASA. (**E**) HB.

RMSD reflects the fluctuation degree of protein conformation and can evaluate the binding stability between ligand and target protein; a smaller value indicates more stable binding [47]. Figure 7A shows that the RMSD of BCL2-Cichorioside B fluctuated within 0.3~0.7 nm in the first half of the simulation and stabilized within 0.4~0.6 nm after 30 ns, indicating the system reached equilibrium; the RMSD of EGFR-Cichorioside B fluctuated within 0.1~0.7 nm and tended to balance (0.5~0.7 nm) after 10 ns. RMSF reflects the flexibility of protein regions; a lower value indicates more stable residues (stronger rigidity), and a higher value indicates more flexible regions (stronger dynamics) [48]. The RMSF of BCL2-Cichorioside B showed significantly higher fluctuations in N-terminal and loop region residues; RMSF of EGFR-Cichorioside B showed significantly higher fluctuations in loop region residues, with an overall greater flexibility (Figure 7B). Rg is used to evaluate the overall compactness of proteins and molecular affinity; a lower value indicates a more stable protein–ligand complex (lower Gibbs free energy and more compact structure). The Rg fluctuation range of all systems was narrow (within 0.1 nm), indicating tight binding between ligands and proteins (Figure 7C). SASA reflects the interaction between proteins and surrounding water molecules, reflecting changes in hydrophilicity and hydrophobicity [47]. Notably, the SASA of the EGFR-Cichorioside B complex was higher than that of BCL2-Cichorioside B (Figure 7D), which is consistent with the hydrophobic interaction characteristics at the protein–ligand interface in molecular docking. HB are key non-covalent interactions that are crucial for stabilizing the three-dimensional structure of protein–ligand complexes. The BCL2-Cichorioside B complex formed an average of 7 hydrogen bonds during the simulation, while the EGFR-Cichorioside B complex formed an average of 4 hydrogen bonds (Figure 7E). BCL2-Cichorioside B had more hydrogen bonds and stronger binding strength, consistent with the molecular docking results.

Taken together, the RMSD, RMSF, Rg, SASA and hydrogen-bond profiles indicate that both complexes reached a locally stable state over the 100 ns trajectory, and the interaction patterns were consistent with the molecular docking results. However, several limitations should be acknowledged. First, the analysis is based on a single 100 ns trajectory per complex; independent replicates were not performed, and therefore no statistical uncertainty can be assigned to the reported observables. Second, 100 ns is a relatively short timescale for fully sampling large-scale conformational transitions, and the present results therefore support the local stability of the docked complexes rather than global conformational convergence. Third, binding free-energy calculations were not performed, so the MD results should be regarded as qualitative supporting evidence for the docking poses rather than as a quantitative measure of binding affinity.

### 2.8. Results of Antioxidant Assays in Vitro

**Figure 8:**
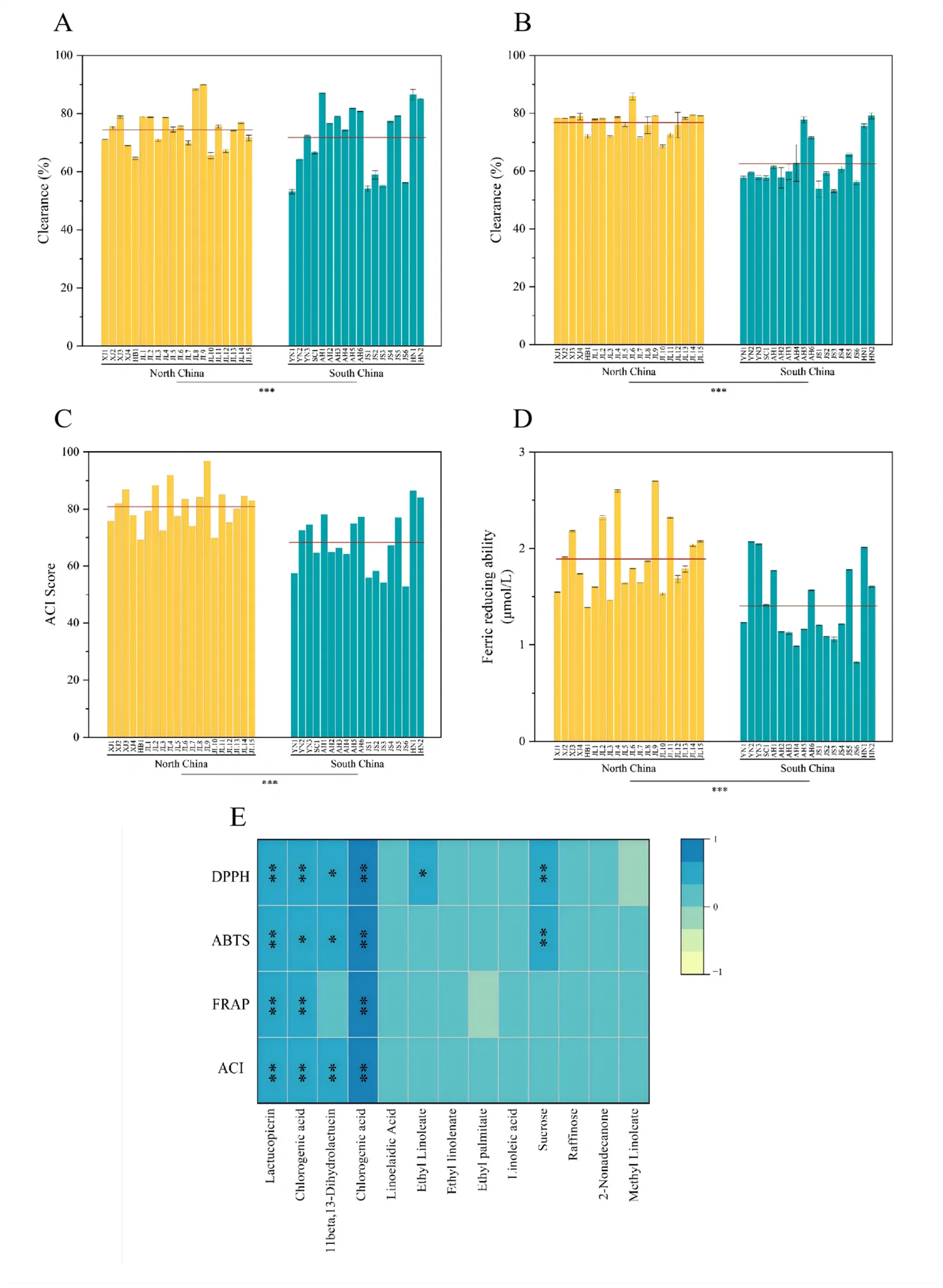
Results of antioxidant assays in vitro. (**A**) DPPH. (**B**) ABTS. (**C**) FRAP. (**D**) ACI. (**E**) Correlation analysis plot between antioxidant capacity and relative contents of 13 antioxidant metabolites. (*: *p* < 0.05; **: *p* < 0.01; ***: *p* < 0.001; The red horizontal line represents the average value of each group of samples.)

To comprehensively evaluate the antioxidant activity of chicory samples, the present study employed DPPH, ABTS, and FRAP assays [20]. As shown, all three methods yielded consistent trends, with extremely significant differences in antioxidant activity between chicory from northern and southern regions (*p* < 0.01) (Figure 8). In the DPPH assay, the average free radical scavenging rate of northern chicory was 74.49%, significantly higher than that of southern chicory (71.80%, *p* < 0.01) (Figure 8A). Among them, the northern sample JL9 showed the highest scavenging rate (89.94%), while the southern sample YN1 exhibited the lowest (53.11%). Results from the ABTS assay indicated that the average free radical scavenging rate of northern chicory was 76.72%, significantly higher than that of southern chicory (62.58%, *p* < 0.01). The northern sample JL6 had the highest scavenging rate (85.80%), followed by JL14 (79.38%), whereas the southern sample JS3 showed the lowest (53.06%) (Figure 8B). Consistent with the DPPH results, the FRAP assay revealed that the average antioxidant capacity of northern chicory was 1.89 μmol·L^−1^, significantly higher than that of southern chicory (1.40 μmol·L^−1^, *p* < 0.01). The northern sample JL9 exhibited the strongest ferric ion reducing capacity (2.70 μmol·L^−1^), while the southern sample JS6 had the lowest (0.82 μmol·L^−1^) (Figure 8C).

Due to the varying affinities of different bioactive compounds in antioxidant assays and potential result deviations caused by differences in the principles of each method, the ACI was used to comprehensively evaluate the antioxidant capacity of extracts [49]. As shown in Figure 8D, the average ACI score of northern chicory was 80.77, significantly higher than that of southern chicory (68.28, *p* < 0.01). Among the northern samples, JL9 had the highest ACI (96.78), followed by JL4 (91.80), JL2 (88.25), and XJ3 (86.72), while the southern sample JS6 had the lowest score (52.75).

Pearson correlation analysis was performed to explore the relationship between antioxidant capacity and the relative contents of 13 antioxidant metabolites identified in Section 3.3.1. The results showed that 7 antioxidant metabolites, including Lactucopicrin, Cichorioside B, 11β,13-Dihydro-8-deoxylactucin, and Chlorogenic acid, were significantly positively correlated with antioxidant capacity. These metabolites, mainly sesquiterpene lactones and phenolic acids, can be regarded as key antioxidant-associated metabolites in chicory. These compositional differences may partly contribute to the higher antioxidant capacity observed in northern chicory; however, the causal contributions of individual metabolites require further targeted validation.

## 3. Materials and Methods

### 3.1. Materials and Reagents

#### 3.1.1. Plant Materials

A total of 38 batches of chicory samples were purchased from the Chongqing Chinese Medicinal Materials Market and local pharmacies in the original producing areas. These samples were identified as the dried roots of *Cichorium intybus* L. by appearance, traits and microscopic identification by Professor Jian Wang from the Chongqing University of Chinese Medicine. All samples were numbered immediately after collection and stored in a cool and dry place in the Analytical Laboratory of the School of Pharmacy, Chongqing Medical University. Specifically, 20 batches were northern samples, consisting of 4 batches from Xinjiang, 1 batch from Hebei, and 15 batches from Jilin; 18 batches were southern samples, including 3 batches from Yunnan, 1 batch from Sichuan, 6 batches from Anhui, 6 batches from Jiangsu, and 2 batches from Henan. An appropriate amount of samples from each producing area was crushed and then passed through an 80-mesh sieve for subsequent determination. Detailed information of the samples is provided in Table S3.

#### 3.1.2. Chemicals and Reagents

Methanol (Chongqing Chuandong Chemical Reagent Co., Ltd., Chongqing, China), acetonitrile (Merck Chemicals (Shanghai) Co., Ltd., Shanghai, China), and formic acid (Shanghai Macklin Biochemical Technology Co., Ltd., Shanghai, China); all the above reagents were of HPLC grade. 1,1-diphenyl-2-picrylhydrazyl (DPPH) assay kit (Beijing Leagene Biotechnology Co., Ltd., Beijing, China), 2,2′-azino-bis (3-ethylbenzothiazoline-6-sulfonic acid) (ABTS) assay kit (Shanghai Adamas Reagent Co., Ltd., Shanghai, China), and Ferric Reducing Antioxidant Power (FRAP) assay kit (Wuhan Elabscience Biotechnology Co., Ltd., Wuhan, China). The instrument used was RRH-A500 High-Speed Multifunctional Grinder (Shanghai Yuanwo Industry and Trade Co., Ltd., Shanghai, China).

### 3.2. Analysis of Volatile Metabolites by HS-GC-MS

Volatile components in chicory were analyzed using a SHIMADZU GCMS-TQ8040 NX system equipped with an AOC 6000 Plus autosampler (Shimadzu Corporation, Kyoto, Japan). Analytical conditions: 0.8 g of powdered sample was accurately weighed into a 20 mL headspace vial, which was heated to 130 °C for 25 min; the temperature of the quantitative loop was 145 °C, and the sampling volume was 1 mL. Chromatographic separation was performed on a SH-Rxi-5Sil MS (30 m × 0.25 mm, 0.25 μm) column (Restek Corporation, Bellefonte, PA, USA). With the following temperature program: initial temperature 50 °C held for 1 min, increased to 100 °C at 2 °C·min^−1^ and held for 2 min, then increased to 140 °C at 10 °C·min^−1^ and held for 2 min, and finally increased to 250 °C at 20 °C·min^−1^ and held for 3 min. The carrier gas was 99.999% helium with a flow rate of 1.0 mL/min and a split ratio of 10:1; both inlet and ion source temperatures were 250 °C. The electron ionization source was operated at 70 eV, with a mass scan range of 45~500 *m*/*z*. Qualitative analysis was performed by matching the National Institute of Standards and Technology (NIST)14 database. No internal standard was used for the volatile analysis. The relative percentage content of each component was calculated by peak-area normalization. This approach provides semi-quantitative relative comparisons among the samples rather than absolute quantification.

### 3.3. Analysis of Non-Volatile Metabolites by UPLC-Q-TOF-MS

#### 3.3.1. Sample Preparation

A total of 0.5 g powdered sample was accurately weighed, mixed with 5 mL 99.90% methanol, and ultrasonically extracted (250 W, 40 kHz) for 30 min. The mixture was centrifuged at 10,000 rpm for 5 min, and the supernatant was filtered through a 0.22 μm membrane. The filtrate was diluted 20-fold for UPLC-Q-TOF-MS analysis.

#### 3.3.2. UPLC-Q-TOF-MS Conditions

Non-volatile components were analyzed using an AB Sciex TripleTOF™ 5600 mass spectrometer (AB Sciex LLC, Framingham, MA, USA). Sample separation was achieved on an ACE Excel 3 SuperC18 (100 mm × 2.1 mm, 3.0 μm) column (Advanced Chromatography Technologies Limited, Aberdeen, Scotland, UK) at 30 °C with a 2 μL injection volume. Mobile phases were 0.1% formic acid aqueous solution (A) and acetonitrile (B) with the gradient: 0~1.2 min, 6% B; 1.2~9.5 min, 6%~75% B; 9.5~11 min, 75%~90% B; 11~13 min, 90% B; 13~15 min, 6% B, at a flow rate of 0.25 mL·min^−1^. QTOF analysis was performed in positive and negative ion modes: capillary voltages 5.0 kV (positive) and 4.5 kV (negative); nebulizing gas, auxiliary gas, and curtain gas at 55, 55, and 25 Psi, respectively; ion source temperature 600 °C; declustering potential 70 V, collision energy 42 eV, collision energy spread 15 eV; scan range 50~1000 *m*/*z*, and detection mode was information-dependent acquisition.

### 3.4. Determination of Content of 5 Bioactive Ingredients

#### 3.4.1. Preparation of the Sample Solution

Batches of chicory samples were pulverized and passed through an 80-mesh sieve and set aside. Accurately weigh 1g of chicory sample powder in a dry 25 mL volumetric flask, add 99.90% methanol to the scale, ultrasonic (250 W, 40 kHz) extraction for 30 min, make up the loss of weight, 0.22 μm membrane filtration of the sample solution, and then follow the same method to prepare a blank solution.

#### 3.4.2. Preparation of the Control Solution

An precise quantity of Chlorogenic acid, Chicoric acid, Isochlorogenic acid B, Isochlorogenic acid A and Isochlorogenic acid C control was measured, resulting in a control stock solution of Chlorogenic acid (620.8 μg·mL^−1^), Chicoric acid (657.6 μg·mL^−1^), Isochlorogenic acid B (500.0 μg·mL^−1^), Isochlorogenic acid A (784.0 μg·mL^−1^) and Isochlorogenic acid C (190.2 μg·mL^−1^) prepared through methanolization in a 10 mL volumetric flask, respectively. Transfer 5 mL of each stock solution of the control mixture into a 50 mL volumetric flask and then dilute with methanol to achieve concentrations of Chlorogenic acid, Chicoric acid, Isochlorogenic acid B, Isochlorogenic acid A and Isochlorogenic acid C at 62.08 μg·mL^−1^, 65.76 μg·mL^−1^, 50.00 μg·mL^−1^, 78.40 μg·mL^−1^ and 19.02 μg·mL^−1^, respectively.

#### 3.4.3. High-Performance Liquid Chromatograph (HPLC) Conditions

Bioactive ingredients were analyzed using a LC-20AT High-Performance Liquid Chromatograph (Shimadzu, Japan). The mobile phases A and B consisted of 0.4% phosphoric acid and methanol, respectively. The gradient elution parameters were as follows: 0~5 min, 10~20% B; 5~15 min, 20~40% B; 15~20 min, 40% B; 20~25 min, 40~65% B; 25~30 min, 65~80% B; 30~40 min, 80~10% B; and 40~45 min, 10% B. The chromatographic column was Thermo BDS C18 (Thermo Fisher Scientific, Waltham, MA, USA) with a detection wavelength of 254 nm, a column temperature of 35 °C, a flow rate at 1 mL·min^−1^, and an injection volume of 10 μL.

### 3.5. Network Pharmacology Prediction

#### 3.5.1. Screening of Active Components and Acquisition of Common Targets

All compounds identified were imported into the TCMSP database https://old.tcmsp-e.com/tcmsp.php (accessed on 1 July 2025) to screen bioactive components using the criteria of oral bioavailability ≥ 30 and drug-likeness ≥ 0.18 [50]. The targets of these active components were retrieved from the Swiss Target Prediction database http://www.swisstargetprediction.ch/ (accessed on 8 July 2025) to obtain target information of the identified key antioxidant bioactive compounds in chicory. The GeneCards https://www.genecards.org/ (accessed on 12 July 2025) and DrugBank https://go.drugbank.com (accessed on 18 July 2025) databases were searched using “oxidative stress” as the keyword. After removing duplicate genes, human antioxidant-related targets were screened with a relevance score > 10. Venn analysis was used to identify the intersection targets between compound targets and antioxidant-related targets. Detailed information of the samples is provided in Table S4.

#### 3.5.2. Construction and Analysis of Protein–Protein Interaction (PPI) Network

The intersection targets were imported into the STRING database https://string-db.org/ (accessed on 28 July 2025), with the species set as “Homo sapiens” to construct a PPI network. The network was imported into Cytoscape 3.8.0 to calculate Degree Centrality (DC), Closeness Centrality (CC), and Betweenness Centrality (BC). Core antioxidant targets with high relevance were screened based on the mean values of these parameters.

#### 3.5.3. Gene Enrichment Analysis

In the R environment, the clusterProfiler, Stringi, and Pathview packages were loaded to perform Gene Ontology (GO) functional enrichment analysis and Kyoto Encyclopedia of Genes and Genomes (KEGG) pathway enrichment analysis on the intersection targets. GO enrichment analysis covered biological processes (BPs), molecular functions (MFs), and cellular components (CCs), with valid results screened at *p* < 0.05.

#### 3.5.4. Molecular Docking

Human protein structures of core antioxidant targets were selected from the Protein Data Bank, and ligand structures were obtained from PubChem. Receptors were prepared by removing crystallographic water and non-essential molecules, followed by hydrogen addition and charge assignment. Ligands were processed using OpenBabel v2.3.1 for hydrogen addition, charge assignment, geometry optimization, and PDBQT conversion. Blind docking was performed using AutoDock Vina 1.2.2 [51,52], with receptor-specific grid boxes covering the receptor and accessible ligand-binding regions. Protein–ligand interactions were visualized using PyMOL 2.5.4. Alternative tautomeric forms were not separately enumerated.

#### 3.5.5. Molecular Dynamics (MD) Simulation

MD simulation of the protein–ligand complexes with the lowest binding energy from molecular docking were performed using Gromacs v2022.03 with the CHARMM36 force field. After energy minimization, equilibration pre-simulations under NVT and NPT ensembles were conducted sequentially, followed by a 100 ns simulation under constant temperature (300 K) and pressure (1 atm). Key dynamic parameters were calculated using Gromacs built-in tools, including Root Mean Square Deviation (RMSD) for evaluating structural stability, Root Mean Square Fluctuation (RMSF) for analyzing residue flexibility, Radius of Gyration (Rg) for assessing compactness, and Solvent-Accessible Surface Area (SASA) for monitoring protein surface properties and ligand binding changes. Interface hydrogen-bond (HB) dynamics were also calculated to characterize interaction stability [53].

### 3.6. In Vitro Antioxidant Assays

#### 3.6.1. DPPH Assay

The DPPH radical scavenging capacity was determined according to the method of Sun et al., with slight modifications [54]. The sample solution used for antioxidant assays was the 20-fold diluted extract described in Section 2.3.1, corresponding to a dry-sample-equivalent concentration of 5 mg/mL. The blank group (*A*_0_) consisted of 100 μL methanol solution and 100 μL DPPH solution; the experimental group (*A*_1_) consisted of 100 μL sample solution and 100 μL DPPH solution; the control group (*A*_2_) consisted of 200 μL sample solution. Thus, the final extract concentration in the DPPH reaction system was 2.5 mg/mL. The reaction mixture was incubated at 25 °C in the dark for 30 min, and absorbance was measured at 510 nm. Results were expressed as radical scavenging activity (%) and calculated using the following equation:

(1) $$Radical\;scavenging\;activity\left(\%\right)=\frac{\left[A_{0}-\left(A_{1}-A_{2}\right)\right]}{A_{0}}\times 100\%$$

#### 3.6.2. ABTS Assay

The ABTS radical scavenging capacity was determined with minor optimization based on the method by Cheng et al. [55]. ABTS stock solution was prepared by mixing equal volumes of ABTS solution and potassium persulfate solution, followed by incubation in the dark for 16 h. Before use, the solution was diluted 40–50 times with methanol to adjust the absorbance to 0.7 ± 0.05 at 734 nm. The blank group (*A*_0_) consisted of 10 μL methanol solution and 190 μL ABTS working solution; the experimental group (*A*_1_) consisted of 10 μL sample solution and 190 μL ABTS working solution; the control group (*A*_2_) consisted of 200 μL sample solution. Thus, the final extract concentration in the ABTS reaction system was 0.25 mg/mL. After incubation at room temperature in the dark for 5 min, absorbance was measured at 734 nm. Results were expressed as free radical scavenging rate (%) using the same formula as Equation (1).

#### 3.6.3. FRAP Assay

FRAP antioxidant activity was assessed following the kit instructions. FeSO_4_ standard solution was prepared to generate a total antioxidant capacity standard curve, and FRAP working solution was freshly prepared according to the kit protocol. The blank group (*A*_0_) consisted of 20 μL methanol solution and 180 μL FRAP working solution; the experimental group (*A*_1_) consisted of 20 μL sample solution and 180 μL FRAP working solution. Thus, the final extract concentration in the FRAP reaction system was 0.50 mg/mL. After incubation at 37 °C for 30 min, absorbance was measured at 593 nm. Results were expressed as ferric reducing ability equivalent to 1.0 μmol/L FeSO_4_, calculated using the formula below:

(2) $$Ferric\;reducing\;ability\;(\mu mol/L)=\frac{A_{1}-A_{0}-b}{a}$$

#### 3.6.4. Antioxidant Capacity Index (ACI)

The ACI was calculated referring to the method described by Wang et al., [21]. The highest value in each assay (DPPH, ABTS, FRAP) was assigned 100 points, and the index score for other samples was calculated as: Score = (Sample score/Best score) × 100. ACI was defined as the mean of the index scores from the three assays.

### 3.7. Statistical Analysis

Multivariate statistical analysis was performed using SIMCA 16 software (Umetrics, Umea, Sweden), employing orthogonal partial least squares-discriminant analysis (OPLS-DA) to evaluate intergroup differences and identify key differential metabolites. All in vitro antioxidant assays were conducted in triplicate. SPSS 27.0 software (SPSS Inc., Chicago, IL, USA) was used to calculate Pearson correlation coefficients for analyzing the relationship between key antioxidant metabolites and antioxidant capacity, with a significance level set at *p* < 0.05. All figures were generated using SIMCA software, R software (version 4.5.1; https://www.r-project.org/ accessed on 3 December 2025), and Origin software (version 2021, OriginLab Inc., Northampton, MA, USA).

#### 3.7.1. Chemometrics

Multivariate statistical analysis was performed using SIMCA 14.1 software (Umetrics, Umea, Sweden). Principal Component Analysis (PCA) was employed for data dimensionality reduction, while OPLS-DA and Variable Importance in Projection (VIP) scores were utilized to evaluate intergroup differences and identify key differential metabolites. All in vitro antioxidant assays were conducted in triplicate. SPSS 27.0 software (SPSS Inc., Chicago, IL, USA) was used to calculate Pearson correlation coefficients for analyzing the correlation between key antioxidant metabolites and antioxidant capacity, with a significance level set at *p* < 0.05. All figures were generated using SIMCA software, R software (Version 4.5.1, https://www.r-project.org/), and Origin software (version 2021, OriginLab Inc., Northampton, MA, USA).

#### 3.7.2. Machine Learning

All algorithms were programmed in Python software (Version 3.10.9, Python Software Foundation, Beaverton, OR, USA), with all adopted algorithms derived from the scikit-learn machine learning standard library. Three algorithms were involved in this study, as detailed below. Logistic Regression (LR) is a linear classification model that transforms real-valued inputs into probabilities ranging from 0 to 1 via the logistic function. A binary LR model can be extended for multi-class classification by adopting the one-versus-rest (OvR) strategy or using cross-entropy as the loss function. Random Forest (RF) is a supervised classification and regression method consisting of multiple decision trees generated by random sampling methods, with all trees being mutually independent. When test samples are fed into the RF model, each decision tree executes a separate classification, and the most frequent classification result across all decision trees is taken as the final classification outcome for the test samples. Support Vector Classification (SVC) is a supervised learning algorithm that identifies an optimal hyperplane for classifying data points to maximize the margin between different classes. It can handle non-linear data through support vectors with the use of kernel functions such as the polynomial and Radial Basis Function (RBF) kernels. In addition, SVC can provide insights into feature importance, particularly when the linear kernel is applied.

In this study, the three aforementioned machine learning models were trained and applied to predict potential chemical biomarkers. Each model generated distinct prediction results, which helped improve the overall accuracy and robustness of the predictive outcomes. By leveraging the inherent advantages of different algorithms, the effective prediction of the potential chemical biomarkers that distinguish north and south chicory samples was achieved.

## 4. Conclusions

This study integrated metabolomics, machine learning, molecular dynamics simulations, and in vitro antioxidant assays to systematically investigate the metabolic profiles and differences in the antioxidant activity of chicory from different regions. A comprehensive characterization of the components in 38 sample batches of chicory from distinct geographical areas was conducted using HS-GC-MS and UPLC-Q-TOF-MS. Chemometrics and machine learning identified 8 volatile and 7 non-volatile potential chemical biomarkers. The present data provide a preliminary chemical and in vitro antioxidant basis for understanding north–south differences in chicory, but bioavailability, safety, stability in food matrices, cellular efficacy, and in vivo effects were not evaluated and require further validation.

This study provides a scientific basis for the development and application of chicory as a natural antioxidant in the food industry, facilitating its practical use in the screening of functional food ingredients and the utilization of natural antioxidant components. In addition, the volatile metabolites were quantified by peak-area normalization without an internal standard; future work should incorporate internal standards for absolute quantification. This research only focused on the analysis of chicory roots and did not cover other organs such as leaves and flowers. Different organs of plants exhibit differences in metabolic profiles and functional activities; thus, using only roots as the research object may not fully reflect the overall antioxidant potential of chicory. Future studies could extend to the systematic comparison of different parts of chicory, providing more comprehensive support for the efficient utilization of the whole plant in the food field.

## Figures and Tables

**Table 1:** Cross-Validation Results.

Dataset	Model	Accuracy (Mean ± SD)
GC (volatile, 64 features)	LR	0.922 ± 0.096
	RF	0.939 ± 0.090
	SVC	0.882 ± 0.124
Q-TOF (non-volatile, 35 features)	LR	0.873 ± 0.101
	RF	0.890 ± 0.112
	SVC	0.937 ± 0.077

## Data Availability

The data are available from the corresponding author.

## Supplementary Materials

The following supporting information can be downloaded at: https://www.mdpi.com/article/10.3390/molecules31203790/s1, Figure S1. The cracking process of representative compounds. (A) Lactucin. (B) Cichorioside K. (C) Cichorioside D. (D) Stachyose. (E) L-Arginine. (F) Chlorogenic acid. (G) 2,3,4,9-Tetrahydro-1H-beta-carboline-3-carboxylic acid. Figure S2. The test of system adaptability and Content of 5 bioactive compounds in chicory from north and south China. (A).The test of system adaptability (1. Chlorogenic acid; 2. Chicoric acid; 3. Isochlorogenic acid B; 4. Isochlorogenic acid A; 5. Isochlorogenic acid C). (B).Content of 5 bioactive compounds in chicory from north and south China. Figure S3. Network pharmacology prediction. (A) “Chicory-metabolite-target” network diagram. (B) Venn diagram. (C) PPI network diagram. (D) GO and KEGG enrichment analysis. Figure S4. Molecular docking between key antioxidant metabolites and core antioxidant targets. (A) Binding energy heatmap. (B) BCL2-Cichorioside B molecular docking diagram. (C) STAT3-Lactucopicrin molecular docking diagram. (D) EGFR-Cichorioside B molecular docking diagram. Table S1. Volatile components in chicory from different origins. Table S2. Linearity, LOD and LOQ of the five components. Table S3. Sample origin and environment information. Table S4 Target information used in network pharmacology analysis.
