# Supplementary material for: Metabolic Profiling, Antioxidant Activity, and Mechanistic Insights of Chicory from Northern and Southern China: Integrating Metabolomics, Machine Learning, Molecular Docking, Molecular Dynamics Simulations, and In Vitro Assays
Source: Molecules. 2026 Oct 21;31(20):3790. doi: 10.3390/molecules31203790 (PMC13707533; doi:10.3390/molecules31203790)
Supplement: Supplementary file 1 [file molecules-31-03790-s001.zip › molecules-4565411-supplementary.pdf]

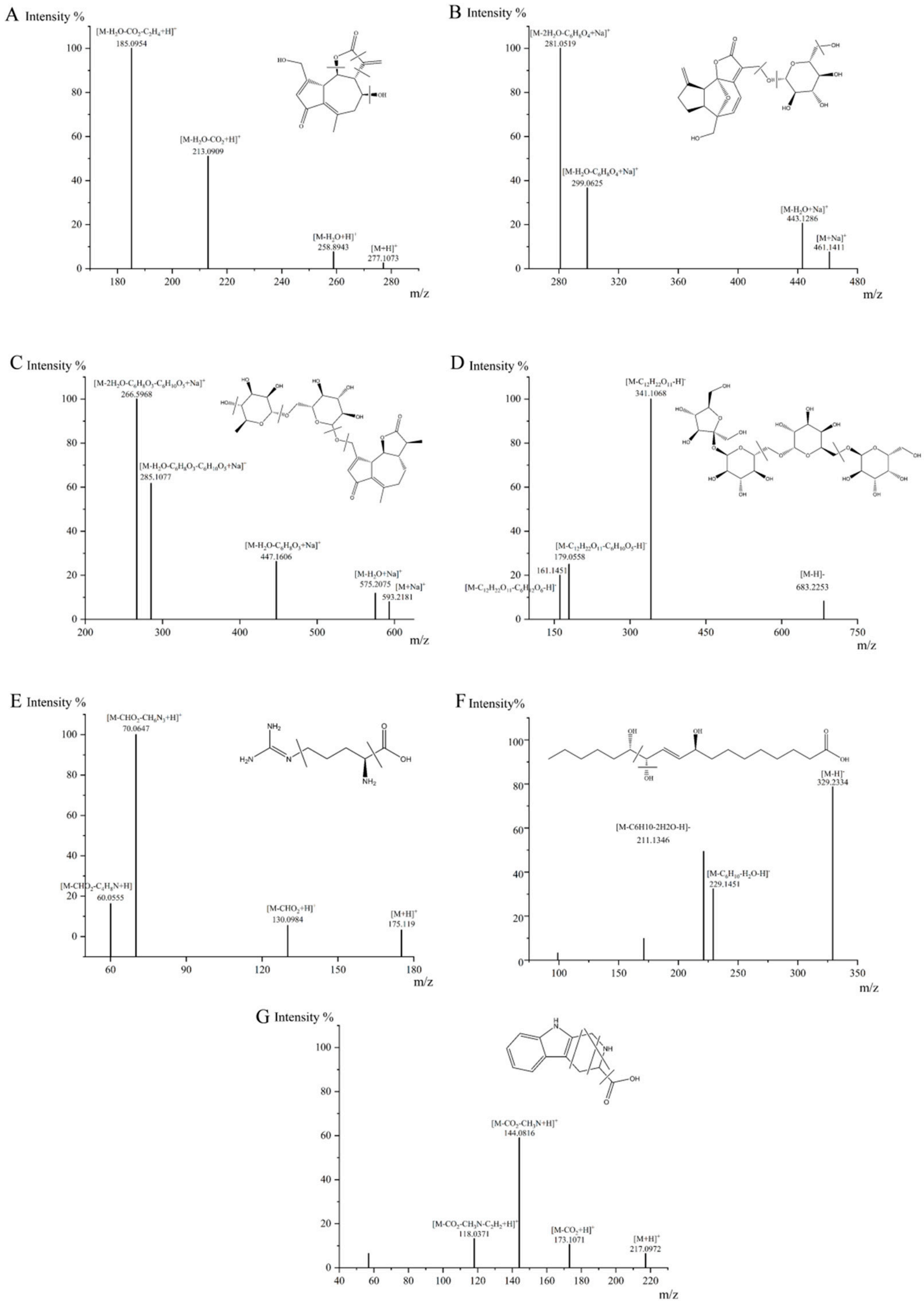

Figure S1 The cracking process of representative compounds. (A) Lactucin. (B) Cichorioside K. (C) Cichorioside D. (D) Stachyose. (E) L-Arginine. (F) Chlorogenic acid. (G) 2,3,4,9-Tetrahydro-1H-beta-carboline-3-carboxylic acid.

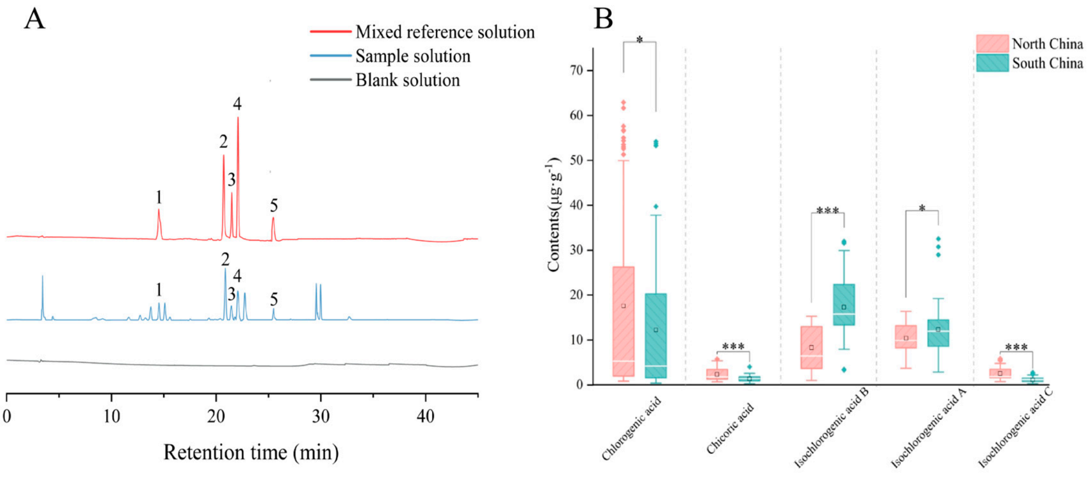

Figure S2. The test of system adaptability and Content of 5 bioactive compounds in chicory from north and south China. (A).The test of system adaptability (1. Chlorogenic acid; 2. Chicoric acid; 3. Isochlorogenic acid B; 4. Isochlorogenic acid A; 5. Isochlorogenic acid C). (B).Content of 5 bioactive compounds in chicory from north and south China.

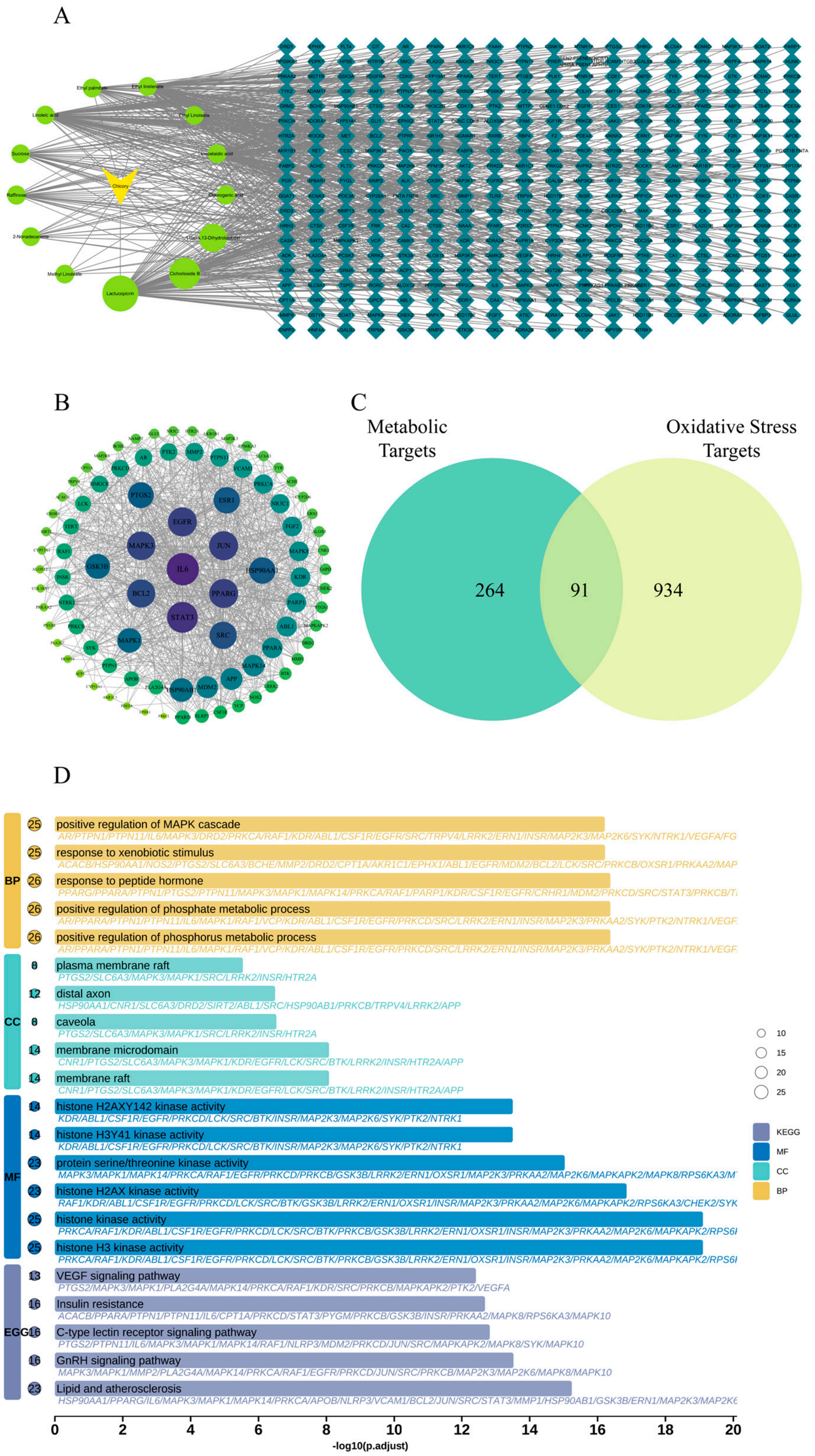

Figure S3. Network pharmacology prediction. (A) "Chicory-metabolite-target" network diagram. (B) Venn diagram. (C) PPI network diagram. (D) GO and KEGG enrichment analysis

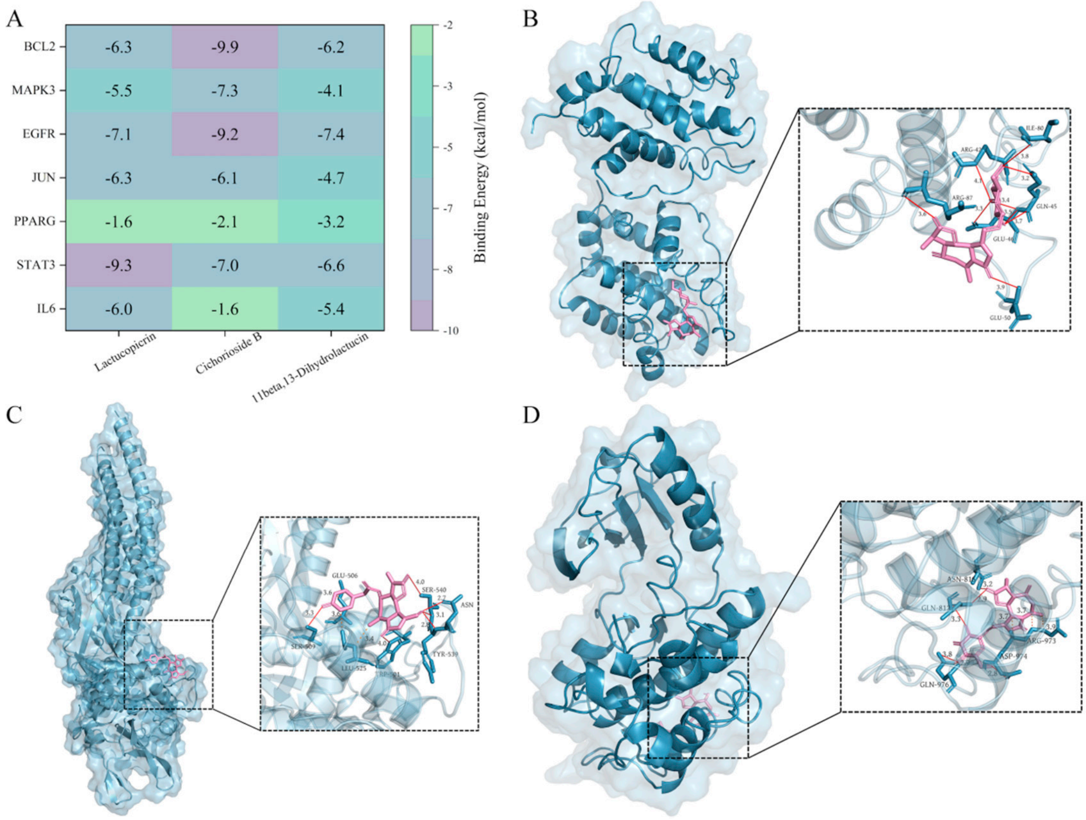

Figure S4. Molecular docking between key antioxidant metabolites and core antioxidant targets. (A) Binding energy heatmap. (B) BCL2-Cichorioside B molecular docking diagram. (C) STAT3-Lactucopicrin molecular docking diagram. (D) EGFR-Cichorioside B molecular docking diagram

Table S1 Volatile components in chicory from different origins.

No.	Compound	Retention time (min)	CAS number	Peak area（10^6)		Odor description
				North China	South China	
	Aldehydes (15)					
1	3-Methylpentanal	3.372	15877-57-3	6.59±40.17	7.86±74.06	
2	Hexanal	3.941	66-25-1	5.71±44.03	11.82±65.22	Fruity, sweaty
3	3-Furaldehyde	4.285	498-60-2	0.07±1.52	0.11±1.53	
4	Furfural	4.642	98-01-1	41.16±206.70	34.22±235.11	Baked bread
5	Heptanal	6.772	111-71-7	0.28±2.02	0.45±2.99	
6	5-Methyl furfural	7.652	620-02-0	0.86±13.63	0.39±2.37	Caramel
7	Benzaldehyde	9.168	100-52-7	4.51±38.37	5.67±25.04	Cherry, almond
8	2-Heptenal	10.174	18829-55-5	0.01±0.42	0.08±0.80	Green vegetable, fresh fatty
9	Octanal	11.315	124-13-0	0.04±0.87	0.16±1.45	Orange peel, green herbal, fresh fatty
10	Pyrrole-2-carboxaldehyde	11.860	1003-29-8	-	0.05±1.05	Coffee
11	Phenylacetaldehyde	13.378	122-78-1	2.95±13.37	3.92±14.05	Honey, cocoa
12	Nonanal	17.184	124-19-6	0.57±2.99	0.95±1.46	Orange peel
13	2-Nonenal	20.649	18829-56-6	0.01±0.62	0.04±0.97	Green cucumber
14	Decanal	23.697	112-31-2	1.10±5.26	1.55±5.22	Orange peel, waxy
15	5-Hydroxymethylfurfural	24.925	67-47-0	2.02±44.73	-	Buttery, waxy
	Ketones (14)					
16	1-Hydroxy-2-butanone	3.598	5077-67-8	0.47±5.23	0.04±1.59	Sweet coffee, butterscotch
17	2-Methyltetrahydrofuran-3-one	4.093	3188-00-9	6.27±35.89	2.44±18.70	Buttery, nutty
18	Acetoxyacetone	5.653	592-20-1	0.74±10.41	0.90±8.82	Buttery, dairy, nutty

19	4-Cyclopentene-1,3-Dione	6.063	930-60-9	0.94±5.60	0.57±5.68	
20	2-Heptanone	6.357	110-43-0	0.17±2.51	0.33±5.65	Coconut, woody
21	2(5H)-Furanone	7.159	497-23-4	0.56±10.51	0.17±7.12	Buttery
22	2,3-Octanedione	8.730	585-25-1	0.08±1.58	1.20±29.92	Herbal, earthy
23	2,4-Dihydroxy-2,5-dimethyl-3(2H)-furanone	10.178	10230-62-3	1.27±16.88	0.47±10.78	
24	2,5-Heptanedione	10.411	1703-51-1	0.02±0.72	0.05±1.12	
25	3,4-dimethylfuran-2,5-dione	15.843	766-39-2	2.03±17.85	0.59±4.98	
26	3-Hydroxy-2-methyl-4-pyrone	17.405	118-71-8	0.78±13.63	-	Sweet, caramel, cotton candy
27	2,3-Dihydro-3,5-dihydroxy-6-methyl-4H-pyran-4-one	19.344	28564-83-2	41.04±484.89	6.48±39.06	
28	3,5-Dihydroxy-2-methyl-4H-pyran-4-one	21.836	1073-96-7	0.94±25.78	-	
29	2-Nonadecanone	37.743	629-66-3	0.02±0.69	0.15±1.45	
	Acids (11)					
30	Isovaleric acid	4.850	503-74-2	3.40±7.35	4.07±7.91	Sour, cheese
31	2-Methylbutanoic acid	5.075	116-53-0	1.01±4.15	1.36±5.61	Cheese
32	Picolinic acid	8.670	98-98-6	0.03±1.05	0.05±1.19	
33	Octanoic acid	10.174	124-07-2	0.57±11.11	0.64±7.44	Rancid, cheese
34	4-Methyloctanoic acid	10.191	54947-74-9	0.38±7.78	0.19±4.41	Musty, creamy milk
35	Caproic acid	10.255	142-62-1	1.35±21.9	1.44±28.81	Sour, cheese
36	Pentanoic acid	10.455	109-52-4	-	0.37±11.11	Acidic, sweaty
37	Pentadecanoic acid	39.463	1002-84-2	-	0.10±3.19	Waxy
38	Palmitic acid	39.470	57-10-3	1.30±17.37	1.59±30.55	Waxy, fatty
39	Linoelaidic acid	40.491	506-21-8	0.04±1.65	0.03±1.27	
40	Linoleic acid	40.497	60-33-3	0.32±7.97	0.85±16.66	Fatty

	Heterocyclics (9)					
41	2-Methylpyrazine	4.547	109-08-0	2.76±7.48	4.98±32.63	Nutty, cocoa
42	2-Acetylfuran	7.012	1192-62-7	6.60±32.81	5.78±39.87	Cocoa, caramel, coffee
43	2,6-Dimethylpyrazine	7.261	108-50-9	4.23±29.96	4.77±71.77	Cocoa, roasted nuts
44	2-Hydroxytetrahydrofuran	10.174	5371-52-8	-	0.07±1.07	
45	2,5-Dimethylpyrazine	10.174	123-32-0	1.32±41.09	3.64±37.93	Cocoa, roasted nuts
46	2-Pentylfuran	10.616	3777-69-3	1.91±6.51	3.84±29.13	Fruity, earthy
47	2-Ethyl-6-methylpyrazine	11.110	13925-03-6	0.05±1.09	0.17±1.71	Roasted potato
48	2-Acetylpyrazine	12.245	22047-25-2	0.01±0.39	0.05±1.06	Popcorn, nutty
49	2-Acetylpyrrole	14.641	1072-83-9	5.98±30.39	3.44±14.6	Walnut, nut
	Esters (7)					
50	4-Butyrolactone	7.195	96-48-0	3.66±45.98	0.22±5.45	Creamy, caramel
51	Furfuryl acetate	10.755	623-17-6	0.08±1.37	0.13±2.47	Fruity, banana
52	Methyl palmitate	39.270	112-39-0	0.16±1.87	0.10±2.09	Oily, waxy
53	Ethyl palmitate	39.649	628-97-7	0.20±3.19	0.31±4.67	Waxy, milky balsam
54	Methyl Linoleate	40.255	112-63-0	0.16±1.92	0.11±2.51	Woody, oily
55	Ethyl Linoleate	40.680	544-35-4	0.40±5.65	0.51±8.95	Fruity, oily
56	Ethyl linolenate	40.725	1191-41-9	0.06±1.10	0.09±2.11	
	Alcohols (5)					
57	1,4-Butanediol	3.162	110-63-4	3.55±22.92	2.46±17.07	
58	Propylene glycol	3.250	57-55-6	2.44±65.98	0.73±14.23	
59	2,3-Butanediol	3.845	513-85-9	5.70±84.61	2.80±46.16	
60	Furfuryl alcohol	5.234	98-00-0	18.97±78.86	13.44±74.02	Caramel, coffee
61	5-Methylfurfuryl alcohol	8.851	3857-25-8	0.48±9.70	0.09±2.50	Sweet caramellic
	Hydrocarbons (3)					
62	Octadec-3-ene	35.389	7206-19-1	0.02±0.78	0.21±3.53	

63	Tetradecane	35.520	629-59-4	-	0.08±1.61	Waxy
64	Nonadecane	36.832	629-92-5	0.03±0.89	0.02±0.77	Bland

Table S2 Linearity, LOD and LOQ of the five components

Compound	Linear equation	R^2	Linear range (μg·mL^{-1})	LOD (μg·mL^{-1})	LOQ (μg·mL^{-1})
Chlorogenic acid	y=243217x-742.93	0.9998	0.6208-62.08	0.0355	0.1242
Chicoric acid	y=125385x-2560.4	0.9998	0.6570-65.70	0.0547	0.1973
Isochlorogenic acid B	y=77834x+1095.2	0.9998	0.5000-50.00	0.0462	0.1667
Isochlorogenic acid A	y=119356x+10182	0.9998	0.7840-78.40	0.0792	0.2354
Isochlorogenic acid C	y=32010x+1942.2	0.9998	0.1902-19.02	0.0571	0.1902

Table S3 Sample origin and environment information

NO.	Collection region	LON(°)	LAT(°)	Group	NO.	Collection region	LON(°)	LAT(°)	Group
XJ1	Xinjiang	126.63	45.61	North China	YN1	Xishuangbanna Yunnan	100.92	22.30	South China
XJ2	Xinjiang	126.63	45.61	North China	YN2	Xishuangbanna Yunnan	100.92	22.30	South China
XJ3	Xinjiang	126.63	45.61	North China	YN3	Xishuangbanna Yunnan	100.92	22.30	South China
XJ4	Xinjiang	126.63	45.61	North China	SC1	Sichuan	104.08	30.63	South China
HB1	Hebei	117.17	39.18	North China	HN1	Xinyang Henan	114.08	32.13	South China
JL1	Changbai Mountains Jilin	120.45	27.78	North China	HN2	Xinyang Henan	114.08	32.13	South China
JL2	Changbai Mountains Jilin	120.45	27.78	North China	AH1	Xuancheng Anhui	118.76	30.94	South China
JL3	Changbai Mountains Jilin	120.45	27.78	North China	AH2	Xuancheng Anhui	118.76	30.94	South China
JL4	Changbai Mountains Jilin	120.45	27.78	North China	AH3	Xuancheng Anhui	118.76	30.94	South China
JL5	Changbai Mountains Jilin	120.45	27.78	North China	AH4	Xuancheng Anhui	118.76	30.94	South China
JL6	Changbai Mountains Jilin	120.45	27.78	North China	AH5	Xuancheng Anhui	118.76	30.94	South China
NO.	Collection region	LON(°)	LAT(°)	Group	NO.	Collection region	LON(°)	LAT(°)	Group
JL7	Changbai Mountains	120.45	27.78	North China	AH6	Xuancheng Anhui	118.76	30.94	South China

	Jilin								
JL8	Changbai Mountains Jilin	120.45	27.78	North China	JS1	Yancheng Jiangsu	120.19	33.33	South China
JL9	Changbai Mountains Jilin	120.45	27.78	North China	JS2	Yancheng Jiangsu	120.19	33.33	South China
JL10	Changbai Mountains Jilin	120.45	27.78	North China	JS3	Yancheng Jiangsu	120.19	33.33	South China
JL11	Changbai Mountains Jilin	120.45	27.78	North China	JS4	Yancheng Jiangsu	120.19	33.33	South China
JL12	Changbai Mountains Jilin	120.45	27.78	North China	JS5	Yancheng Jiangsu	120.19	33.33	South China
JL13	Changbai Mountains Jilin	120.45	27.78	North China	JS6	Yancheng Jiangsu	120.19	33.33	South China
JL14	Changbai Mountains Jilin	120.45	27.78	North China					
JL15	Changbai Mountains Jilin	120.45	27.78	North China					

Table S4 Target information used in network pharmacology analysis.

Table S4A Predicted metabolite targets of chicory bioactive compounds (n = 355).

Target symbol	Target symbol	Target symbol	Target symbol	Target symbol	Target symbol	Target symbol
CES1	CES2	NR1I3	CA2	CA1	CA4	AR
CYP19A1	ACACB	PAOX	MGLL	CTSS	CTSL	HSP90AA1
PPARG	PPARA	PPARD	FABP4	FABP3	FFAR1	PTGS1
FABP5	FAAH	TERT	FABP1	SCD	CNR1	PTPN1
ALOX5	POLB	PTPN2	PTGES	LTB4R	ESR2	PTPN6
CDC25A	RORC	HSD11B1	HMGCR	TOP1	PTPRF	AKR1B10
PDE4D	PTGER2	ALOX12	CDC25B	NR3C1	NOS2	NR1H3
PTGIR	HSD11B2	PTGDR2	TRPV1	PTGS2	CD81	PRKCH
CYP26B1	CYP26A1	ESR1	ACP1	SERPINA6	SHBG	G6PD
CYP51A1	PTGER4	FNTA FNTB	PLA2G1B	SRD5A2	ADORA3	PTPN11
NPC1L1	SIGMAR1	CYP17A1	NR3C2	FFAR4	IL6	GLUL
PREP	PTGER1	RXRB	PGR	SLC16A1	SLC6A3	MAPK3
ENPP2	PSEN2 PSENEN NCSTN APH1A PSEN1 APH1B	GRM2	RBP4	GPBAR1	HTR2B	RORB
MAPK1	HNF4A	ITGAL ICAM1 ITGB2	BCHE	PDE4A	MMP2	TOP2A
PLA2G4A	PRKAG1 PRKAB1 PRKAA2	TRPM8	CMA1	CTSG	RORA	ALOX15
DRD2	ADRA2B	MAPK14	ALOX5AP	FABP2	PDE4B	UGT2B7
VDR	PAM	ACHE	HSD17B3	SIRT2	PRKCA	SOAT1
SOAT2	RAF1	PRKCE	PRKCQ	PRKCG	CPT1A	PARP1
PTPN13	PDE10A	PPM1B	PPP1CC	VCP	GRIN2B	F2R
CNR2	ADORA2A	NPY5R	AKR1C2	AKR1C1	FLT1	KDR
HSD17B2	MTTP	APOB	PFKFB3	HRH3	HRH4	ATIC
CCNE1 CDK2	AVPR2	CASR	AVPR1A	QPCT	EPHX1	ROCK2
ROCK1	CTSK	NLRP3	ABL1	VCAM1	CSF1R	PDGFRB
KIT	FLT4	PDGFRA	EGFR	RET	MAP3K20	FRK
PTK6	DDR1	CIT	CDK8	CDK19	MAP3K19	GCGR
SLC9A1	SLC9A2	CRHR1	MDM4	NAAA	MDM2	AKR1C3

MET	BRPF1	P2RX7	NAMPT	TRIM24	SMO	LIMK2
MMP13	PRKCD	TSPO	JAK1	PLA2G7	MCL1	BCL2
DGAT1	KCNK3	KCNK9	CSNK1A1	CSNK1D	PLK1	PDE5A
C5AR1	KCNA3	IMPDH2	GRM5	SLC5A2	MTNR1A	MTNR1B
PRCP	PDE3A	PPP2R5A	JUN	ADORA1	ATP2A1	SLC29A1
TYR	GLI1	IARS	GLRA1	GLRA2	PPP2CA	IGFBP3
EPHA2	EPHX2	LCK	SRC	MAP3K9	FGFR1	AURKA
SLC5A1	BTK	STAT3	KDM3A	MMP1	KDM5B	MMP14
MMP8	KDM4D	KDM4C	F2	PYGL	PYGM	ADK
PIM1	LGALS3	LGALS9	HSP90AB1	TLR9	PRKCB	VAV1
TRPV4	ABCB1	PLA2G2A	GSK3B	PGGT1B FNTA	PCSK7	TYK2
MKNK2	ALK	EPHB4	CCR1	MMP3	ADAM17	CCNC CDK8
CDC42BPA	LRRK2	GRK7	STK38	HIPK4	TAOK2	ERN1
OXSR1	MAK	STK39	CDKL5	CDKL3	PRPF4	PIK3C2G
MAP3K6	MAP3K13	ICK	MAP3K15	MAST1	SBK1	HUNK
CDK13	FYN	CASK	PRPF4B	DSTYK	MAP3K12	IGF1R
FLT3	INSR	YES1	MAP2K3	PRKAA2	JAK3	MAP2K6
AURKB	MAPKAPK2	BLK	MAPK8	RPS6KA3	PHKG2	MYLK
AKT2	DAPK3	CAMK1	CAMK4	CHEK2	PDPK1	RPS6KA1
DAPK1	PRKD3	PRKCI	SYK	CSK	MAPK10	MST1R
PTK2	MAP3K11	FGFR3	MYLK2	CAMK2D	ZAP70	NTRK1
GSK3A	MAP3K10	PRKG2	CDK1	VEGFA	FGF1	HPSE
FGF2	LGALS4	LGALS8	ADRA2A	ADRA2C	DRD1	ADRA1D
HTR2A	HTR2C	DRD3	CYP2D6	HTR6	ADRA1A	HTR1B
FOLH1	AMY1A	AKR1B1	MMP12	APP		

Table S4B Intersecting targets between predicted metabolite targets and antioxidant-related targets used for PPI and enrichment analyses (n = 91).

Target symbol	Target symbol	Target symbol	Target symbol	Target symbol	Target symbol	Target symbol

AR	ACACB	HSP90AA1	PPARG	PPARA	PPARD	PTGS1
TERT	CNR1	PTPN1	ALOX5	HMGCR	ALOX12	NR3C1
NOS2	PTGS2	ESR1	ACP1	G6PD	CYP51A1	PTPN11
CYP17A1	NR3C2	IL6	GLUL	SLC6A3	MAPK3	MAPK1
BCHE	MMP2	PLA2G4A	DRD2	MAPK14	ACHE	SIRT2
PRKCA	RAF1	CPT1A	PARP1	VCP	AKR1C1	KDR
APOB	EPHX1	NLRP3	ABL1	VCAM1	CSF1R	EGFR
CRHR1	MDM2	NAMPT	PRKCD	PLA2G7	BCL2	PDE5A
JUN	TYR	LCK	SRC	BTK	STAT3	MMP1
PYGM	HSP90AB1	PRKCB	TRPV4	GSK3B	LRRK2	ERN1
OXSR1	INSR	MAP2K3	PRKAA2	MAP2K6	MAPKAPK2	MAPK8
RPS6KA3	MYLK	CHEK2	PRKCI	SYK	MAPK10	PTK2
NTRK1	VEGFA	FGF2	HTR2A	CYP2D6	AKR1B1	APP
